# USP10 deubiquitinates RUNX1 and promotes proneural-to-mesenchymal transition in glioblastoma

**DOI:** 10.1038/s41419-023-05734-y

**Published:** 2023-03-22

**Authors:** Wenjin Qiu, Zumu Xiao, Yushi Yang, Lishi Jiang, Shibin Song, Xiaolan Qi, Yimin Chen, Hua Yang, Jian Liu, Liangzhao Chu

**Affiliations:** 1 Department of Neurosurgery, The Affiliated Hospital of Guizhou Medical University, Guiyang, 550001 Guizhou China; 2 Department of Pathology, The Affiliated Hospital of Guizhou Medical University, Guiyang, Guizhou China; 3 Key Laboratory of Endemic and Ethnic Diseases, Ministry of Education & Key Laboratory of Medical Molecular Biology of Guizhou Province, Guizhou Medical University, Guiyang, Guizhou China; 4 Department of Neurosurgery, Guizhou Provincial People’s Hospital, Guiyang, 550001 Guizhou China

**Keywords:** Tumour biomarkers, Oncogenes

## Abstract

The mesenchymal (MES) subtype of glioblastoma (GBM) is a highly aggressive, malignant and proliferative cancer that is resistant to chemotherapy. Runt-related transcription factor 1 (RUNX1) was shown to support MES GBM, however, its underlying mechanisms are unclear. Here, we identified USP10 as a deubiquitinating enzyme that regulates RUNX1 stabilization and is mainly expressed in MES GBM. Overexpression of USP10 upregulated RUNX1 and induced proneural-to-mesenchymal transition (PMT), thus maintaining MES properties in GBM. Conversely, USP10 knockdown inhibited RUNX1 and resulted in the loss of MES properties. USP10 was shown to interact with RUNX1, with RUNX1 being stabilized upon deubiquitylation. Moreover, we found that USP10 inhibitor Spautin-1 induced RUNX1 degradation and inhibited MES properties in vitro *and* in vivo. Furthermore, USP10 was strongly correlated with RUNX1 expression in samples of different subtypes of human GBM and had prognostic value for GBM patients. We identified USP10 as a key deubiquitinase for RUNX1 protein stabilization. USP10 maintains MES properties of GBM, and promotes PMT of GBM cells. Our study indicates that the USP10/RUNX1 axis may be a potential target for novel GBM treatments.

## Introduction

Glioblastoma multiforme (GBM) is the most common and aggressive primary malignancy of the adult central nervous system (CNS) [1, 2]. Current standard treatments for GBM patients include surgical resection, postoperative concurrent radiotherapy, and chemotherapy. However, such treatments have not significantly improved the prognosis of GBM patients, with a dire 15–month median survival and a 5-year survival rate less than 5% [3]. Previous studies have identified three subtypes of GBM, mesenchymal (MES), classical (CL), and proneural (PN) [4]. Among these, MES is considered the most malignant subtype of GBM and closely connected to poor survival and prognosis. Interestingly, it was observed that PN subtypes can differentiate into MES [4–6]. Similar to epithelial-mesenchymal transition (EMT) in other cancers, the PN-to-MES transition (PMT) is a key event driving glioma invasion, malignant progression, and poor prognosis [7–9]. Nevertheless, the underlying mechanisms of PMT in GBM are still unclear and need to be explored.

Runt-related transcription factor 1 (RUNX1) is a promising candidate to regulate PMT [10]. RUNX1 belongs to the RUNX family, which contains a conserved Runt domain responsible for sequence-specific DNA binding [11]. This transcription factor exhibits seemingly contradictory effects in different cancers, since RUNX1 has been implicated as a tumor suppressor gene in solid tumors (e.g., breast and gastric cancer) [12, 13], while it functions as an oncogene in colorectal, ovarian, head and neck cancers [10]. In GBM, RUNX1 promotes the invasion of tumor cells, and correlates with the maintenance of the MES phenotype and poor prognosis of GBM patients [14, 15]. However, the underlying mechanisms by which RUNX1 promotes invasion and maintain MES remain largely unknown. The RUNX1 protein is regulated by post-translational modifications (PTMs), such as the ubiquitin proteasome pathway-mediated degradation, which is connected to the activity of RUNX1 [16]. On the other hand, it is unclear which processes regulate RUNX1 deubiquitination, a reverse process of ubiquitination [14, 15]. Therefore, the stability of RUNX1 in GBM cells and the molecular mechanisms that stimulates PMT progression remain to be clarified.

Deubiquitinases (DUBs), as the name implies, regulate the ubiquitin signaling network and control a myriad of important cellular proteins [17]. Indeed, ubiquitin specific proteases (USPs), which are a subtype of DUBs, comprise nearly 70 members and were shown to stabilize target proteins [18–20]. The role of USPs in various diseases, especially malignancies, has been reported by numerous studies [21]. USPs are important in tumor development and progression, regulating invasion, migration, proliferation, stemness, drug resistance and tumor-associated microenvironment of tumor cells, including glioblastoma [22, 23]. As a member of DUBs in mammalian cells, ubiquitin-specific peptidase 10 (USP10) plays multiple roles in tumors by regulating different substrates [24, 25]. For example, circWSB1 is induced by HIF1α under hypoxic conditions and could bind USP10 to disrupt the interaction between USP10 and p53, which leads to the poly-ubiquitination and subsequent degradation of p53, thus promoting the progression of breast cancer [26]. Additionally, decreased expression of USP10 or combined USP10/p14ARF is a strong indicator of poor prognosis in patients with ovarian cancer [27]. Moreover, USP10 expression in gastric carcinoma tissues was lower than that in non-cancerous mucosa tissues, and was an independent prognostic factor for the overall survival in gastric carcinoma [28]. Conversely, increased USP10 expression is closely associated with proliferation and metastasis in several tumors, such as non-small-cell lung cancer, chronic myeloid leukemia, colorectal cancer, prostate cancer, and hepatocellular carcinoma [24, 29–33], and is significantly correlated with a poor prognosis of GBM patients [29]. In addition, elevated cell apoptosis and significant cell cycle arrest are observed in the GBM cells after USP10 expression was silenced [25]. However, it is unclear whether USP10 partakes in the molecular network that regulates RUNX1 in GBM.

In this study, we report that USP10 is upregulated in GBM. Such upregulation led to the deubiquitination and stabilization of RUNX1 expression in GBM cells, thereby promoting PMT. Our results reveal that the USP10/RUNX1 axis is a potential target for therapeutic strategies against the malignant progression of GBM.

## Materials and methods

### Cell lines and culture

Human glioblastoma cell lines U251, U87MG, T98G and LN229 were purchased from the American Type Culture Collection (ATCC). Primary cell lines that derived from GBM surgical specimens were maintained in primary serum-free cultures grown on laminin-coated plates. The HEK293T cells were obtained from Bena Culture Collection Technology (China). All cells were cultured in FBS (10%)- and antibiotics-contained Dulbecco’s modified Eagle’s medium (DMEM) at 37 °C. The NHAs human astrocytes were maintained per the manufacturer’s instructions. All cells were routinely tested for mycoplasma contamination bimonthly using MycoAlert PLUS Kits (Lonza).

### Antibodies and reagents

The following monoclonal antibodies were used: USP10 (ab109219, Abcam), RUNX1 (ab240639, Abcam), FLAG-tag (cat#14793, Cell Signaling Technology), His-tag (cat#12698, Cell Signaling Technology), HA-tag (cat#3724, Cell Signaling Technology), YKL-40 (#AF2599, Novus Biologicals), Olig2 (cat# sc-293163, Santa Cruz), PDGFRα (cat# 3174, Cell Signaling Technology), MET (cat# sc-8057, Santa Cruz), COL5A1 (cat# 86903, Cell Signaling Technology) and β-actin (ab8227, Abcam). Spautin-1 (cat# 17769), an inhibitor of USP10, was supplied by Cayman. Cycloheximide (CHX, cat# C7698), a specific inhibitor for protein translation, was purchased from Sigma. MG132 (cat# S2619), the proteasome inhibitor, was supplied by Selleckchem.

### Tissue samples

10 normal brain tissues (NBTs) and 58 glioblastoma samples were obtained from the Affiliated Hospital of Guizhou Medical University. NBTs were collected from patients with traumatic brain injury undergoing craniotomy decompression. 36 glioblastoma samples exhibited high expression of MES GBM marker (YKL-40, MET, and COL5A1) and low expression of PN GBM marker (Olig2, PDGFRα). Two neuropathologists identified the histological specimens independently according to the WHO criteria. In Supplementary Table. S1, we summarized the clinicopathological characteristics collected from patients enrolled in this study. All procedures of experiments using human samples were strictly reviewed and approved by the Institute Research Medical Ethics Committee of Guizhou Medical University. All patients enrolled in this study signed consent forms before the initiation of this study.

### RNA extraction and qRT-PCR analysis

RNA extraction and qRT-PCR analysis were performed as previously described [34]. The following primers were used for qRT-PCR: USP10, 5’-TTTTAAATGCCACCGAACCTATC-3’ (forward) and 5’-CCAGCCATTCAGACCGATCT-3’ (reverse); RUNX1, 5’-CACTGTGATGGCTGGCAATGATG-3’ (forward) and 5’-CTCTGTGGTAGGTGGCGACTTG-3’ (reverse); YKL-40, 5’- GAAGACTCTCTTGTCTGTCGGA-3’ (forward) and 5’- AATGGCGGTACTGACTTGATG-3’ (reverse); Olig2, 5’-GAATCCGCTGGTATCCACGA-3’ (forward) and 5’-GCGGAGCGAGACGTTTAGAA-3’ (reverse); PDGFRα, 5’-TAGTGCTTGGTCGGGTCTTG-3’ (forward) and 5’- TTCATGACAGGTTGGGACCG-3’ (reverse); MET, 5’- AGCAATGGGGAGTGTAAAGAGG-3’ (forward) and 5’-CCCAGTCTTGTACTCAGCAAC-3’ (reverse); COL5A1, 5’- TACCCTGCGTCTGCATTTCC-3’ (forward) and 5’- GCTCGTTGTAGATGGAGACCA-3’ (reverse). Relative expression mRNAs levels were normalized to that of GAPDH and calculated by the standard 2^-ΔΔCt^ method.

### Transfection

Two non-overlapping lentiviral human USP10 shRNA sequences (shUSP10#1 sequence: 5’-CCTATGTGGAAACTAAGTATT-3’; shUSP10#2 sequences: 5’-CCCATGATAGACAGCTTTGTT-3’) were cloned into pLKO.1/U6 (Addgene). Flag-tagged USP10, mutant C424A (a dominant negative form) USP10, and His-tagged RUNX1 (NP_001745.2, 480 a.a. coding sequence) were cloned into indicated plasmids and verified by DNA sequencing. Plasmids containing HA-tagged ubiquitin, ubiquitin-Lys48 (pRK5-HA-ubiquitin-Lys48), and ubiquitin-Lys63 (pRK5-HA-ubiquitin-Lys63) were supplied by Addgene. A QuikChange Mutagenesis Kit (Agilent Technologies) was employed for site-directed mutation of USP10. The cells were seeded on a 6-well plate and incubated for 24 h, and were used for transfection after reaching 70–80% confluence. All transfections used Lipofectamine 3000 (Invitrogen, catalog L3000150) and were performed following the manufacturer’s instructions. Stable cell lines following transfection were screened by puromycin.

### Transwell invasion and migration assays

To further assess invasiveness using transwell assay, the cells were seeded in the upper chambers at the density of 2 × 10^4^ cells per well in serum-free medium, and the lower chambers were filled in DMEM supplemented with 10% FBS to stimulate invasion. After 24 h of incubation, the upper chamber cells were removed using a cotton swab, and the membrane was fixed in 4% paraformaldehyde for 15 min and stained with crystal violet for 15 min. Five fields of adherent cells in each well were photographed randomly. To measure migration, the filters were not pre-coated with Matrigel.

### CCK-8 assay

The proliferation rate of glioblastoma cells was examined by the CCK-8 kit (Beyotime) following the manufacturer’s instructions. Briefly, transfected cells were seeded into 96-well plates at the density of 5 × 10^3^ cells/well, and 10 µl CCK-8 reagent was added per well at 24, 48, 72, and 96 h of culture. The absorbance at 450 nm was measured to calculate the percentage of viable cells. The test was repeated three times for each biological replicate.

### Western blot

As previously described [35], Western blot was used to assay the target proteins expression. Cellular proteins were lysed by RIPA buffer containing protease inhibitors (Roche). Cell lysates were subjected to SDS-PAGE and then electrotransferred onto polyvinylidene difluoride membranes, followed by incubation with the indicated primary antibodies overnight. The membranes were then washed, and probed with HRP-conjugated secondary antibodies. Signals were captured on films (Amersham Hyperfilm) and digitized using an optical scanner (Epson V850).

### siRNA library screening

siRNA library screening was performed as previously described [34]. Briefly, the human deubiquitinating enzyme siGENOME RTF Library was purchased from Dharmacon. The screen was performed according to manufacturer’s instructions. Individual siRNA in the DUB siRNA library was transiently transfected into HEK293T cells at a final concentration of 100 nM. 48 h later, cell lysates were extracted and the expression of endogenous RUNX1 was examined by Western blot.

### Immunohistochemistry (IHC)

Immunohistochemistry (IHC) experiments were performed as previously reported [36]. Paraffin-embedded tissue sections (4-µm-thick) were baked at 60 °C for 1 h, deparaffinized in xylene, and rehydrated in graded concentrations of ethanol (100, 95, and 85% for 5 min each). The sections were placed in pH 6.0 citric buffer for 20 min, treated with 3% hydrogen peroxide in phosphate-buffered saline, and incubated with goat serum. Each section was incubated with primary and HRP-secondary antibodies separately. DAB-based chromogen (Vector laboratories) was added and the specimens were counterstained with hematoxylin. Based on staining intensity, we scored the expression of USP10 and RUNX1 from 0 to 3 under a ×40 magnification objective.

### Co-IP assay

NETN buffer containing protease inhibitors was used to lyse cells subjected to different transfections. After incubation with protein A/G agarose beads (Santa Cruz) and indicated primary antibodies at 4 °C, the cell lysates were rinsed four times using NETN buffer. Immunoprecipitated complexes were then subjected to 10% SDS-PAGE gel electrophoresis and immunoblotting analysis.

### GST pulldown assay

Bacterial-expressed GST or GST-RUNX1 bound to glutathione-Sepharose 4B beads (GE Healthcare) were incubated for 2 h at 4 °C with the cell lysate of HEK293T cells expressing Flag-USP10 (WT) or Flag-USP10 (C424A). Then, after being washed at least 4 times with GST-binding buffer, the complexes were detected by immunoblot.

### Deubiquitination assays

Briefly, after HA-ubiquitin transfection and 12-hour treatment with MG132, cells were lysed in RIPA buffer and incubated with IgG or anti-RUNX1 antibody at 4 °C overnight. After immunoprecipitation of endogenous RUNX1 or HA-ubiquitinated His-RUNX1, the ubiquitination level of RUNX1 was detected by immunoblot.

### In vivo xenografts

For intracranial xenograft studies, male nude mice (5-6 weeks of age) were randomly divided into the corresponding groups (*n* = 10/group). 5 × 10^5^ luciferase-expressing U251, LN229, GBM1 and GBM2 cells with indicated modification were intracranially injected into the right caudate nucleus/dorsal striatum of immunocompromised mice using a stereotactic apparatus (coordinates: 2 mm anterior, 2 mm lateral, 3 mm depth from the dura). In vivo bioluminescence imaging was used to monitor tumor growth. Four to ten weeks after GBM cells implantation, the mice were humanely killed and their brains were harvested, paraffin embedded, stained with hematoxylin and eosin (H&E) to confirm the presence of tumors, and subjected to immunohistochemical staining. For the animal survival analysis, mice were maintained until manifestation of pathological symptoms (i.e., hunched back, loss of body weight, reduced food consumption, and inactivity) from tumor burden developed or 70 days after injection. For testing in vivo inhibition effect of USP10 inhibitor Spautin-1, 5 × 10^5^ luciferase-expressing LN229 and GBM2 cells were implanted intracranially. ALZET micro-osmotic pumps (DURECT Corp.) and infusion apparatus were implanted into tumor-bearing mice, either Spautin-1 (20 mg/kg) or vehicle was initiated. Tumor growth was monitored by bioluminescent imaging and mice were maintained until the manifestation of neurologic signs. Samples were coded for analysis so that the experimenter is blinded to experimental grouping. All animal experiments were conducted according to the approved protocol by the Animal Welfare Ethical Review Committee.

### Statistical analysis

All values are presented as mean ± s.e.m. Sample sizes and number of independent experiments were estimated by power analyses using G*Power, which takes pre-specified effect size, type I and II errors as input arguments. Shapiro–Wilk test and F test were first used to test normality and equal variance. Student’s *t* test (two groups) or one/two-way analysis of variations (ANOVA, > two groups) were used for normal-distributed/equal variance data. A nonparametric Mann–Whitney U test was used for non-normally distributed data. For normally distributed data, outliers were defined as >3 standard deviations from the mean and were excluded from analysis. Pearson correlation was applied to assess the correlation between the levels of USP10 and RUNX1 expression. The Kaplan–Meier method was used for the survival analysis of GBM patients and mice models. Data visualization and statistical analysis were conducted using GraphPad Prism 9 software. *p* < 0.05 was considered statistically significant.

## Results

### Identification of USP10 as a potential DUB of RUNX1

**Fig. 1:**
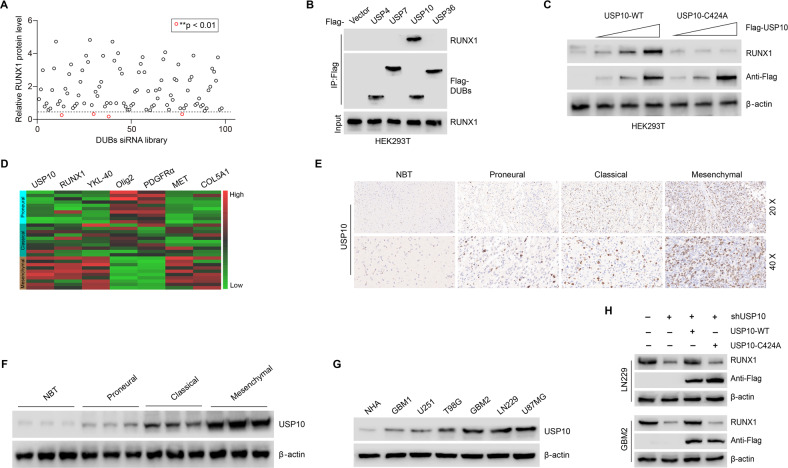
USP10 deubiquitinates RUNX1, which is highly expressed in GBM and positively correlates with MES features. **A** DUB siRNA library screening revealed that siRNA-mediated inhibition of multiple DUB genes decreased RUNX1 protein levels. **p < 0.01. **B** Four Flag-tagged DUBs (USP4, USP7, USP10, and USP36) were expressed in HEK293T cells, and cell lysates were analyzed by IP with Flag beads followed by IB with antibodies against RUNX1 and Flag. **C** Increasing amounts of USP10-WT or USP10-C424A were transfected into HEK293T cells and RUNX1 expression was detected by Western blot. **D** Heatmap showing the gene expression levels of *USP10*, *RUNX1* and markers of PN and MES in different GBM samples (*n* = 30 cases). **E** Expression of USP10 in NBTs and PN, CL, and MES subtypes of GBM tissues detected by IHC. **F** USP10 protein levels in NBTs and PN, CL, and MES subtypes of GBM samples detected by Western blot. **G** USP10 protein levels in NHA, four GBM cell lines and two primary GBM cells. **H** RUNX1 protein levels in LN229 and GBM2 cells transfected with plasmids expressing USP10-WT or USP10-C424A.

RUNX1 function is controlled by multiple regulatory mechanisms, including PTMs. Ubiquitination-driven proteolytic degradation of RUNX1 is a central PTM that regulates RUNX1 stability and activity [16]. Through removing ubiquitin from target proteins, DUBs can effectively reduce protein ubiquitination and degradation levels [19]. To identify DUBs associated with the stabilization of RUNX1 protein, a siRNA library, which could specifically silence 98 DUBs, was constructed and screened. The results showed that USP4, USP7, USP10, and USP36 were significantly connected to the expression of RUNX1 (Fig. 1A and Supplementary Fig. S1A). However, only the interaction between USP10 and RUNX1 was observed (Fig. 1B), thus implicating USP10 in the regulation of RUNX1. Next, we constructed the Flag-tagged USP10-WT (wild-type) and mutant USP10 (C424A, which is a dominant negative form that lost its DUB activity) [37] plasmids and transfected them into HEK293T cells. We found that ectopic expression of USP10-WT, but not USP10-C424A, resulted in RUNX1 protein elevation in a dose-dependent manner in HEK293T cells (Fig. 1C), reiterating the potential role of USP10 in stabilizing RUNX1 expression through its DUB activity.

### USP10 is highly expressed in GBM and positively correlated with MES features

According to data from GEPIA [38], GBM tissues have a higher expression of USP10 and RUNX1 (*n* = 163) in comparison to that of NBTs (*n* = 207) (Supplementary Fig. S2A). Moreover, we analyzed USP10 protein levels in 58 grade IV GBM tissues and 10 normal tissues, and found that GBM tissues exhibited a dramatically higher expression of USP10 (Supplementary Fig. S2B). Indeed, previous studies have shown that RUNX1 is a mesenchymal-related marker, and is upregulated in MES GBM cell lines and tissues [14]. To clarify the link between USP10 and RUNX1 in GBM subtypes, we constructed a heatmap for the expression markers of GBM-associated tumor markers across GBM types. YKL-40 (a chitinase-like glycoprotein coded by *CHI3L1* gene), MET (mesenchymal-epithelial transition protein, a tyrosine kinase receptor), COL5A1 (collagen type V alpha 1) were selected as MES subtype markers, Olig2 (oligodendrocyte transcription factor 2), PDGFRα (platelet-derived growth factor receptor alpha) were PN subtype markers [9, 39, 40]. Positive and negative associations of the expression of USP10 with MES (RUNX1, YKL-40, MET, and COL5A1) and PN (Olig2, PDGFRα) markers were observed (Fig. 1D).

Next, we examined USP10 expression levels in NBTs and in MES, CL, and PN subtypes through IHC. The expression of USP10 in NBTs was lower than that in GBM tissues, with the lowest expression in PN and the highest expression in MES among the three subtypes of GBM (Fig. 1E). In line with above observations, Western blot confirmed that MES subtype samples had higher protein levels of USP10 (Fig. 1F). To further investigate the association between USP10, RUNX1, and markers of PN and MES, we compared USP10 protein levels in GBM cell lines and NHAs. We tested molecular markers across four established and two primary GBM cell lines. U251 and T98G cell lines and primary GBM1 cells were considered to be PN subtypes with higher expression of Olig2 and PDGFRα, while LN229, U87MG cell lines and primary GBM2 cells were considered MES subtypes with higher expression of MES markers (YKL-40, MET, and COL5A1) [9, 39] (Supplementary Fig. S2C). In comparison to NHAs, the MES GBM cells (GBM2, LN229, and U87MG) exhibited a dramatically higher USP10 expression, while the PN GBM cells (T98G, U251, and GBM1), showed only slightly increased USP10 expression (Fig. 1G). Furthermore, compared to control LN229 and GBM2 cells, USP10-deficient cells (using small hairpin interference RNA knockdown) exhibited significantly reduced levels of RUNX1, which could be effectively restored by USP10-WT overexpression, but not USP10-C424A (Fig. 1H).

### RUNX1 knockdown inhibited USP10-induced PMT in GBM

**Fig. 2:**
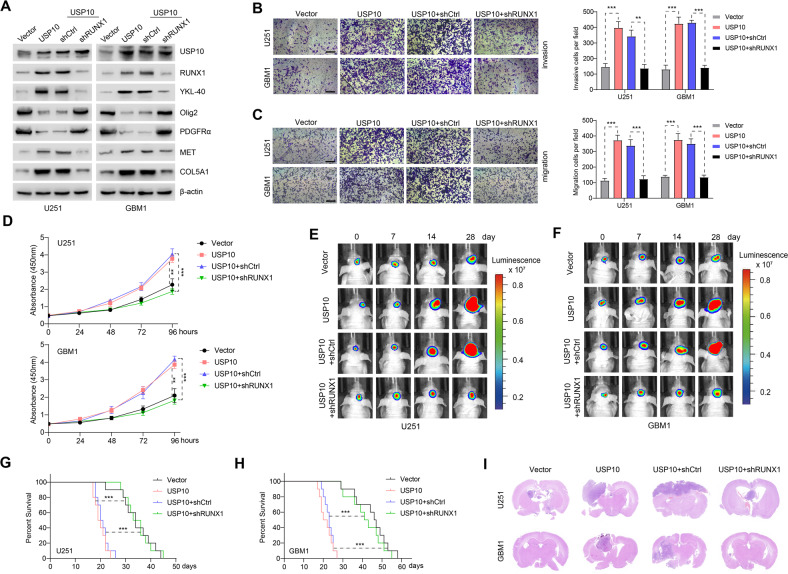
Knockdown of RUNX1 inhibited USP10-induced PMT in GBM. **A** Levels of USP10, RUNX1, PN markers (Olig2 and PDGFRα) and MES markers (YKL-40, MET, and COL5A1) in U251 and GBM1 cells expressing control vector/USP10 or shCtrl/shRUNX1. Transwell assays were used to assess cell invasion (**B**) and migration (**C**) after indicated treatments. Scale bar: 100 μm. *Right* panel shows the quantification. **D** Proliferation of indicated cells. **E**, **F** Representative images showing the in vivo bioluminescence of GBM. Photons/s/cm^2^/steradian was represented by colored scale bars. Each group contains 10 mice. **G**, **H** Representative Kaplan–Meier survival curves showing the survival rate of indicated groups of mice. **I** Representative images of HE staining of intracranial xenografts. **p < 0.01; ***p < 0.001.

To explore USP10-dependent mechanisms underlying the invasion, migration, proliferation, and tumorigenesis of GBM cells, we used U251 and GBM1 cells to evaluate whether USP10 overexpression affected PN and MES markers. Upon USP10 upregulation, MES markers (YKL-40, MET, and COL5A1) were increased, while PN markers Olig2 and PDGFRα were decreased. These changes in PMT biomarkers were reversed when RUNX1 was knocked down (Fig. 2A). This led us to hypothesize that the effects of USP10 on PMT were dependent on RUNX1. As such, we performed transwell assays and CCK8 to assess the effects of USP10 on the development of GBM. We found that USP10 overexpression dramatically enhanced the invasive, migrative, and proliferative capacities of U251 and GBM1 cells, and that such effects were antagonized by RUNX1 knockdown (Fig. 2B–D). Next, we assessed the effect of USP10 in an orthotopic xenograft model. Bioluminescence images revealed that significant differences in tumor volume between U251 and GBM1 cells overexpressed USP10 displayed a significant induction of tumor growth compared with xenografts transduced with vector, and the effects were reversed by RUNX1 knockdown (Fig. 2E, F and Supplementary Fig. S3A). These findings were further confirmed by the survival curves, in which USP10-overexpressing xenografts exhibited significantly decreased survival compared with relevant control, the effects were again reversed by RUNX1 knockdown (Fig. 2G, H). Histologically, H&E staining showed that the USP10-overexpressing xenografts displayed rampant tumor growth, whereas this effect was reversed by inhibition of RUNX1 (Fig. 2I). Furthermore, in comparison to the controls, the expression of MES markers was markedly upregulated, while PN markers were downregulated in USP10-overexpressing xenografts, an effect that was antagonized by RUNX1 knockdown (Supplementary Fig. S3B). These results demonstrated that USP10 overexpression may up-regulate RUNX1 and induce PMT in GBM cells both in cell lines and under in vivo conditions.

### RUNX1 overexpression reversed the suppressive effect of USP10 knockdown on PMT in GBM

**Fig. 3:**
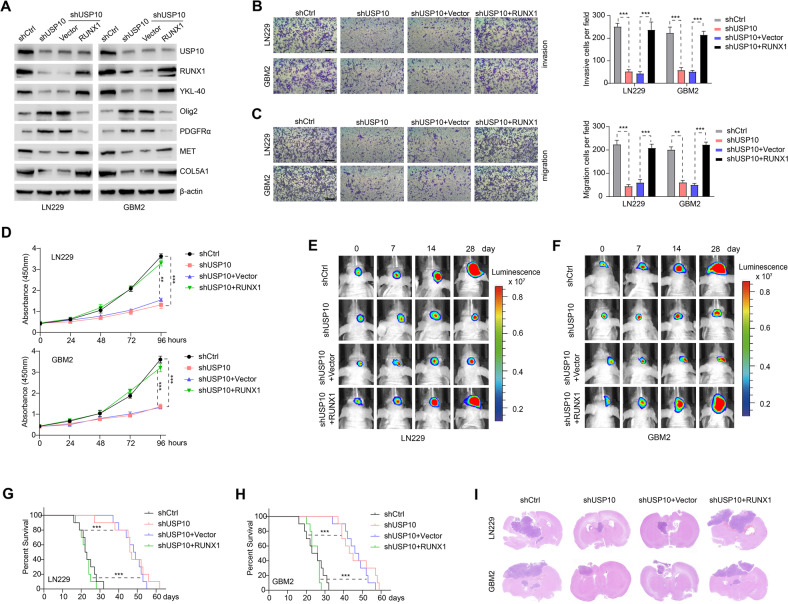
Overexpression of RUNX1 reversed the inhibitory effect of USP10 knockdown on PMT in GBM. **A** Expression of USP10, RUNX1, PN markers (Olig2 and PDGFRα) and MES markers (YKL-40, MET, and COL5A1) in LN229 and GBM2 cells expressing shCtrl/shUSP10 or control vector/RUNX1. Transwell assays were used to assess invasion (**B**) and migration (**C**) of indicated cells. Scale bar: 100 μm. *Right* panel shows the quantification results. **D** CCK8 assays showing cell growth after indicated treatments. Representative images showing the in vivo bioluminescence of GBM induced by LN229 (**E**) and GBM2 (**F**) cells subjected to different treatments. Photons/s/cm^2^/steradian is represented by colored scale bars. Each group contains 10 mice. **G**, **H** Representative Kaplan–Meier survival curves showing the survival rate of indicated groups of mice. **I** Representative images of the HE staining of intracranial xenografts. **p < 0.01; ***p < 0.001.

We next investigated whether RUNX1 overexpression may antagonize the effects of USP10 loss of function. Knockdown of USP10 in LN229 and GBM2 cells resulted in downregulation of MES markers (YKL-40, MET, and COL5A1), while PN markers Olig2 and PDGFRα were increased. Importantly, these changes were reversed by RUNX1 overexpression (Fig. 3A). Additionally, the inhibition of USP10 significantly impaired the invasive, migrative, and proliferative capacities of LN229 and GBM2 cells, an effect that were also reversed by RUNX1 overexpression (Fig. 3B–D). Furthermore, mice bearing USP10-depleted LN229 and GBM2 cells had a delayed tumor formation compared with relevant control, and these alterations were reversed by RUNX1 overexpression (Fig. 3E, F and Supplementary Fig. S4A). Kaplan–Meier survival curve showed that the mice injected with USP10-depleted LN229 and GBM2 cells lived much longer than those injected with control shRNA, and the survival rates in mice intracranially injected with USP10-depleted LN229 and GBM2 cells significantly decreased after RUNX1 overexpression (Fig. 3G, H). The results of H&E staining indicated that the xenografts carrying USP10 shRNA LN229 and GBM2 cells displayed restricted tumor growth, whereas this effect was reversed by overexpression of RUNX1 (Fig. 3I). Furthermore, in comparison to controls, USP10-deficient tumors exhibited significantly reduced expression of MES markers and enhanced expression of PN markers, and these alterations were reversed by RUNX1 overexpression as well (Supplementary Fig. S4B). These observations further support that loss of USP10 may inhibit RUNX1 and impair PMT in GBM cells.

### USP10 interacts with RUNX1

**Fig. 4:**
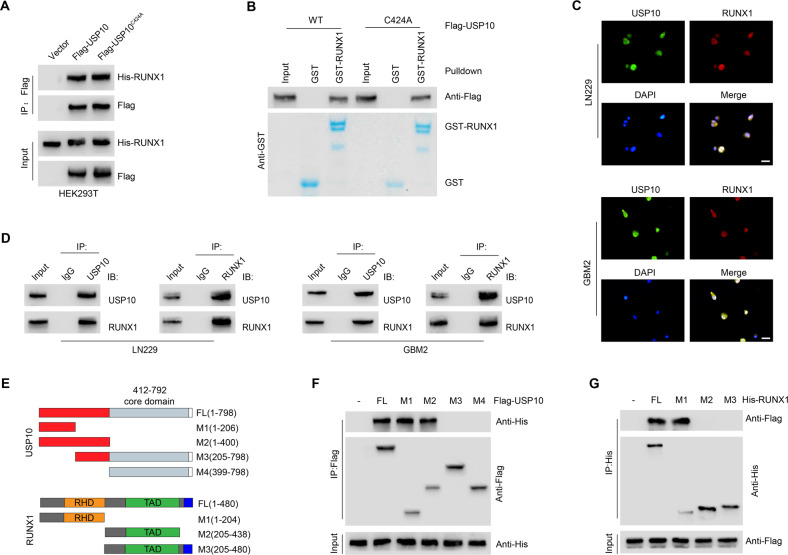
USP10 interacts with RUNX1. **A** Interaction between His-RUNX1 and Flag-tagged USP10 WT or USP10 C424A was confirmed by coimmunoprecipitation in HEK293T cells. **B** Purified Flag- USP10 WT or USP10 C424A was incubated with GST or GST-RUNX1 coupled to glutathione-Sepharose beads in HEK293T cells. Proteins retained on Sepharose were then subjected to IB with indicated antibodies. **C** Representative confocal images showing colocalization of USP10 (green) and RUNX1 (red) in LN229 and GBM2 cells. Scale bars, 10 μm. **D** Coimmunoprecipitation showing the interaction between USP10 and RUNX1 in LN229 and GBM2 cells. **E** Constructs of Flag-tagged full-length (FL) USP10, His-tagged FL RUNX1, and their various deletion mutants. **F** Coimmunoprecipitation confirming the interaction between His-RUNX1 and Flag-tagged FL USP10 or its indicated mutants in HEK293T cells. **G** Coimmunoprecipitation confirming the interaction between Flag-USP10 and His-tagged FL RUNX1 or its indicated mutants in HEK293T cells.

To clarify the possible interaction between USP10 and RUNX1, we performed co-immunoprecipitation (Co-IP). The interaction of RUNX1 with USP10-WT or USP10-C424A was observed (Fig. 4A). Next, GST pull-down assay demonstrated that GST-RUNX1 can be directly bound to USP10-WT or USP10-C424A, but not to GST alone (Fig. 4B), indicating that DUB activity of USP10 is not required for its interaction with RUNX1. Immunofluorescence staining showed that USP10 extensively colocalized with RUNX1 in the nucleus of LN229 and GBM2 cells (Fig. 4C). Furthermore, a direct physical interaction between USP10 and RUNX1 was also identified endogenously in both cell lines (Fig. 4D). To further assess which structural domains of USP10 and RUNX1 mediate this interaction, we mutated the regions that are potentially associated with the interaction with RUNX1 in USP10 (Fig. 4E). We found that the interaction of USP10 with RUNX1 was controlled by the (1–206) N-terminal fragment of USP10 (Fig. 4F). These results indicate that the regulatory domain of USP10 located in the N-terminal is necessary for its binding of RUNX1, as no interaction was observed after the deletion of this regulatory domain. Additionally, our results also showed that the N-terminal sequence of RUNX1, which contains a Runt domain (amino acids 1–204), is required for the direct interaction with USP10 (Fig. 4G).

### USP10 stabilizes RUNX1 through deubiquitination

**Fig. 5:**
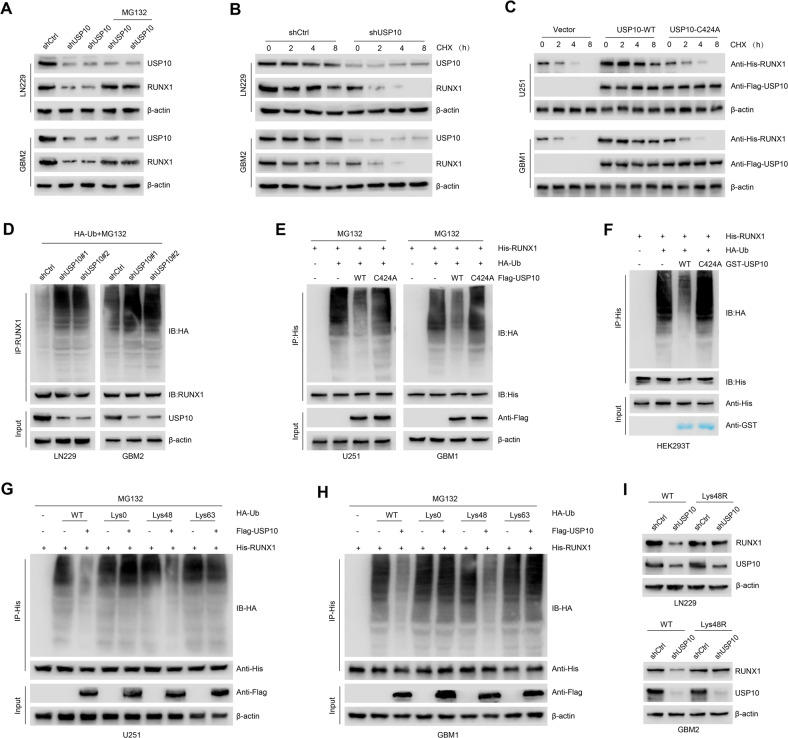
USP10 stabilizes RUNX1 through deubiquitination. **A** Expression of USP10 and RUNX1 in LN229 and GBM2 cells after different treatments. **B** Western blot showing the expression of indicated proteins in LN229 and GBM2 cells after indicated treatments. **C** Expression of indicated proteins in U251 and GBM1 cells after different treatments. **D** LN229 and GBM2 cells were co-transfected with HA-Ub, ShCtrl or shUSP10 and cell lysates subjected to IP with RUNX1 antibody, followed by IB with antibodies against HA and RUNX1. Cells were treated with 20 μM MG132 for 8 h**. E** U251 and GBM1 cells were co-transfected with His-RUNX1, HA-Ub, and Flag-USP10 WT or USP10 C424A and cell lysates were subjected to IP with His antibody, followed by IB with indicated antibodies. Cells were treated with 20 μM MG132 for 8 h. **F** Unubiquitinated or ubiquitinated His-RUNX1 was incubated with GST-USP10 WT or GST-USP10 C424A coupled to glutathione-Sepharose beads. Proteins retained on Sepharose were then subjected to IB with indicated antibodies. Recombinant GST-USP10 WT or GST-USP10 C424A was analyzed by SDS-PAGE and Coomassie blue staining. U251 (**G**) and GBM1 (**H**) cells were co-transfected with His-RUNX1, Flag-USP10, and the indicated HA-Ub Lys0, Lys48-only, or Lys63-only plasmids, RUNX1 ubiquitylation linkage was analyzed. **I** LN229 and GBM2 cells co-transfected with Ub-WT or Ub-Lys48R and shCtrl or shUSP10, RUNX1 levels were analyzed.

In comparison to control cells, we did not observe changes in RUNX1 mRNA expression in USP10-silenced LN229 and GBM2 cells (Supplementary Fig. S5A). This suggests that USP10 is likely to regulate RUNX1 at the posttranslational level. Additionally, these USP10-deficient cells exhibited a reduced RUNX1 expression that was restored by the addition of the proteasomal inhibitor MG132 (Fig. 5A). Next, the protein synthesis inhibitor cycloheximide was used to confirm the regulatory effect of USP10 on RUNX1 stability in control and USP10-deficient LN229/GBM2 cells. We observed that the degradation of RUNX1 was positively correlated to the reduction of USP10 expression (Fig. 5B and Supplementary Fig. S5B). Enforced expression of USP10-WT, but not USP10-C424A, resulted in a prominent increase in the stability of ectopically expressed RUNX1 protein in U251 and GBM1 cells (Fig. 5C and Supplementary Fig. S5C). Next, the ubiquitination level of RUNX1 was determined. We knocked down USP10 in LN229 and GBM2 cells using two non-overlapping lentiviral shRNAs. We noted that a significantly elevated ubiquitination of RUNX1 in USP10-silenced compared to that of control cells (Fig. 5D). Moreover, we observed a significantly reduced ubiquitination of RUNX1 in USP10-overexpressing U251 and GBM1 cells, but not in cells overexpressing mutant C424A-USP10 (Fig. 5E).

To further confirm that RUNX1 is a direct deubiquitinated substrate of USP10, we incubated polyubiquitinated RUNX1 with purified GST-USP10 WT or GST-USP10 C424A under cell-free conditions. The de-ubiquitination of RUNX1 was affected by the incubation of polyubiquitinated RUNX1 with GST-USP10 WT, but not GST-USP10 C424A (which is nevertheless able to interact with RUNX1) in vitro (Fig. 5F), suggesting that USP10 directly removes ubiquitin from RUNX1. It was previously shown that the Lys48- and Lys63-linked ubiquitination of substrate proteins can mediate protein degradation through proteasome- and endosomal–lysosomal-dependent-pathways, respectively [23, 41]. Our observations indicate that USP10 exerts its effect on RUNX1 by removing the ubiquitin linked to Lys48, not Lys63 (Fig. 5G, H). Furthermore, we expressed an Lys48- resistant (Lys48R) form of ubiquitin in USP10 inhibition LN229 and GBM2 cells and found that enforced expression of Lys48R ubiquitin reduced USP10 inhibition–induced RUNX1 downregulation (Fig. 5I). Taken together, these data provide strong evidence that USP10 stabilizes RUNX1 through deubiquitination.

### Inhibition of USP10 by small molecule inhibitors leads to RUNX1 degradation and inhibits PMT

**Fig. 6:**
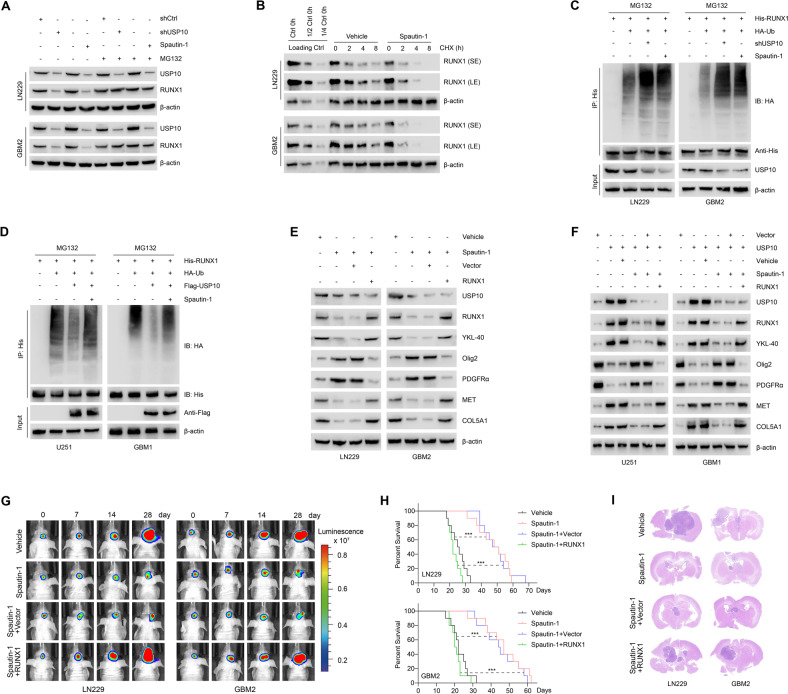
Inhibition of USP10 by small molecule inhibitors leads to RUNX1 degradation and inhibition of PMT. **A** RUNX1 expression in LN229 and GBM2 cells with indicated treatments. **B** LN229 and GBM2 cells were treated with vehicle or 1 μM Spautin-1 for 24 h, followed by 100 μg/ml CHX, harvested at indicated times, and then subjected to IB with antibodies against RUNX1. SE short exposure, LE long exposure. **C** LN229 and GBM2 cells were co-transfected with His-RUNX1, HA-Ub and shUSP10 in the absence or presence of 1 μM Spautin-1, cell lysates were subjected to IP with His antibody, followed by IB with indicated antibodies. Cells were treated with 20 μM MG132 for 8 h. **D** U251 and GBM1 cells were co-transfected with His-RUNX1, HA-Ub and Flag-USP10 in the absence or presence of 1 μM Spautin-1, cell lysates were subjected to IP with His antibody, followed by IB with indicated antibodies. Cells were treated with 20 μM MG132 for 8 h. **E** Western blot showing levels of USP10, RUNX1, PN markers (Olig2 and PDGFRα) and MES markers (YKL-40, MET, and COL5A1) in LN229 and GBM2 cells treated with vehicle or 1 μM Spautin-1, reconstituted with vector control or RUNX1. **F** Western blot showing the levels of USP10, RUNX1, PN markers (Olig2 and PDGFRα) and MES markers (YKL-40, MET, and COL5A1) in U251 and GBM1 cells. **G** Representative in vivo bioluminescent images of intracranial GBM xenografts derived from LN229 and GBM2 cells following treatment with 20 mg/kg Spautin-1 or vehicle, reconstituted with vector control or RUNX1. Colored scale bars represent photons/s/cm^2^/steradian. *n* = 10 each group. **H** Kaplan–Meier survival curves of tumor-bearing mice in indicated groups. **I** Representative images of HE-stained intracranial xenografts. ***p < 0.001.

Since USP10 plays a key role in GBM PMT progression, we used the selective USP10 small molecule inhibitor Spautin-1 to investigate the USP10-RUNX1 axis [24, 31]. We found that Spautin-1 inhibited the expression of USP10 and RUNX1 proteins, without affecting mRNA levels (Supplementary Fig. S5D). Such an inhibitory effect on RUNX1 was reversed by the inhibition of proteasome using MG132 (Fig. 6A). Indeed, combined treatment of Spautin-1 and CHX could significantly reduce the half-life of RUNX1 protein to less than 2 h (Fig. 6B). The effect of Spautin-1 on RUNX1 protein ubiquitination degradation was similar to that of USP10 knockdown in LN229 and GBM2 cells (Fig. 6C). Spautin-1 almost completely inhibited the ability of USP10 to deubiquitinate RUNX1 in U251 and GBM1 cells (Fig. 6D), while this inhibitor reduced the expression of RUNX1 and MES subtype markers and increased the expression of PN subtype markers in GBM cells. These effects were reversed by the overexpression of RUNX1 (Fig. 6E, F). Furthermore, Spautin-1 inhibited the invasion, migration and proliferation of LN229 and GBM2 cells, which was also abrogated by RUNX1 overexpression (Supplementary Fig. S6A–C).

Next, we treated mice harboring intracranial tumors derived from luciferase-expressing LN229 and GBM2 cells with 20 mg/kg Spautin-1. Compared with vehicle-treated mice, tumor-bearing mice receiving Spautin-1 showed inhibited tumor formation and growth rate, which was reversed by RUNX1 overexpression (Fig. 6G and Supplementary Fig. S6D). Kaplan–Meier survival curve revealed that the mice treated with Spautin-1 lived significantly longer compared with vehicle-treated mice, and the enhanced survival rates was reversed by RUNX1 overexpression (Fig. 6H). H&E staining showed that Spautin-1 inhibits intracranial tumor formation, an effect that was also reversed by RUNX1 overexpression (Fig. 6I). Immunohistochemical analysis showed that Spautin-1 attenuated the expression levels of USP10, RUNX1 and MES markers and enhanced PN markers in xenograft tumors of nude mice (Supplementary Fig. S6E). The above findings indicate that USP10 inhibition by the small molecule inhibitor Spautin-1 may promote the degradation of RUNX1 to inhibit PMT, thereby inhibiting GBM progression.

### Clinical relevance of the USP10/RUNX1 axis in GBM

**Fig. 7:**
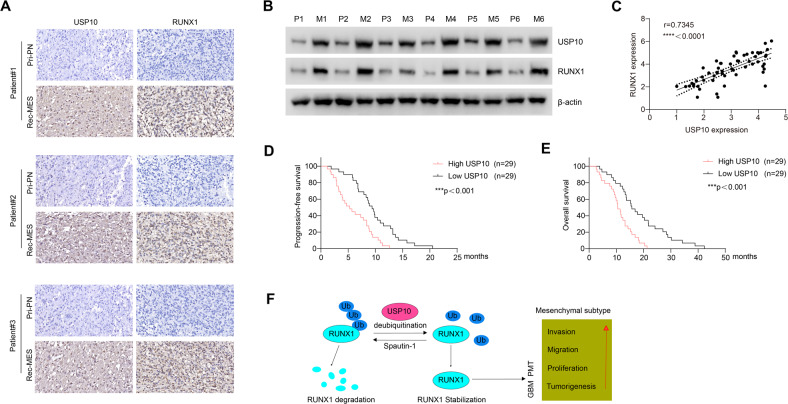
Clinical relevance of USP10/RUNX1-driven PMT in human GBM. **A** Representative images showing the expression of USP10 and RUNX1 in two matched pairs of relapsed MES and primary PN tumors. **B** Western blot showing USP10 and RUNX1 levels in primary PN tumors and corresponding relapsed MES tumors. **C** Correlation between USP10 and RUNX1 in GBM tissues. Progression-free (**D**) and overall (**E**) survival of GBM patients with different USP10 expression. **F** Schematic illustration of main findings of the study, which supports USP10-mediated RUNX1 stabilization promotes proneural-to-mesenchymal transition (PMT) in GBM. ***p < 0.001; ****p < 0.0001.

To further assess the clinical importance of our findings, USP10 and RUNX1 expressions were measured in primary GBM specimens (PN subtype) and corresponding recurrent GBM specimens (MES subtype). In comparison to primary PN tumors, MES tumors showed significantly elevated levels of USP10 and RUNX1 (Fig. 7A and Supplementary Fig. S7A). In parallel there was a dramatic elevation in USP10 and RUNX1 protein levels in recurrent MES GBM tissues, in comparison to primary PN tumor ones (Fig. 7B). A total of 58 GBM specimens were analyzed by immunostaining. We observed a significantly positive correlation between the expression of USP10 and RUNX1 (Fig. 7C). Indeed, patients with a high USP10 expression (*n* = 29) exhibited a worse disease-free survival and overall rate in comparison to patients with low USP10 expression (*n* = 29) (Fig. 7D, E). Collectively, these results demonstrate that USP10 can significantly induce the activation of RUNX1, and the USP10/RUNX1 molecular axis significantly affects clinical outcomes of GBM by promoting PMT in GBM (summarized in Fig. 7F).

## Discussion

PMT is an EMT-like process in GBM that is closely related to GBM invasion, proliferation, poor prognosis, tumor recurrence, and chemotherapy resistance [7, 8]. This phenomenon is relevant to intrinsic cell factors, pro-inflammatory cytokines, radiotherapy, chemotherapy, and anti-angiogenesis therapy [42, 43]. Some molecular effectors involved in PMT have been reported, but the specific molecular mechanism remains to be uncovered.

RUNX1 contributes the migration, invasion, angiogenesis and MES subtype of GBM, and is related to the prognosis of glioma patients [14, 15, 44]. Thus, mechanisms regulating RUNX1 expression represent a promising molecular target for the development of GBM targeted therapy. Due to multiple PTMs, such as ubiquitination, methylation, acetylation, and phosphorylation, RUNX1 is unstable in cells [16]. The ubiquitin proteasome pathway mediates the proteolytic degradation of RUNX1. For instance, the ubiquitin ligase STUB1 regulates the stability and activity of RUNX1 in leukemia [45]; RNF38 promotes RUNX1 ubiquitination and enhances RUNX1-mediated erythroid transcriptional program inhibition [46]; while FZR1 regulates ubiquitin-dependent degradation of RUNX1 in aplastic anemia [47]. The reverse process of ubiquitination is defined as de-ubiquitination, and underlying mechanisms of RUNX1 de-ubiquitination regulation are completely unknown. Here, we identified USP10 as a deubiquitinating enzyme that regulates RUNX1 de-ubiquitination.

The effect of USP10 in different tumors is context-dependent. USP10 was known to be associated with the prognosis of GBM patients – indeed, USP10 knock down in GBM cells can induce apoptosis [25, 29]. However, the biological function of USP10 is understudied in GBM. There are three subtypes of GBM, namely PN, CL and MES, among which the MES subtype is the most malignant [6]. Our data confirmed that USP10 is a key inducer of PN phenotype conversion into MES. In comparison to normal brain tissues, we found a significantly higher expression of USP10 in GBM, especially in the MES subtype. Moreover, we showed that USP10 knockdown in MES GBM cells resulted in loss of MES properties and decreased tumorigenic ability, while overexpression of RUNX1 reversed the inhibitory effect of USP10 knockdown. In addition, our study showed that targeting USP10 can inhibit PMT, thus uncovering a potential novel treatment strategy for GBM. Although multiple deubiquitinating enzymes are difficult targets for targeted therapy (partly due to their high activity and overlapping substrates) [19, 48], we found that a novel small molecule inhibitor targeting USP10, Spautin-1, which promoted the polyubiquitination degradation of RUNX1, showed robust efficacy in preclinical models, setting the stage for the development of a pharmacological inhibitor of USP10.

The ubiquitin proteasome pathway-mediated protein degradation controls the stability of RUNX1 in cells. We found that USP10 regulated the protein level of RUNX1, but had no effect on its mRNA level. These results suggest that the protein level of RUNX1 is regulated by USP10 and that this regulation occurs via post-translational modification, not via gene transcription. USP10 does not activate RUNX1 gene expression to promote the increase in the RUNX1 protein levels; instead, USP10 most likely deubiquitinates RUNX1 and inhibits RUNX1 degradation by the 26S proteasome, thus maintaining the RUNX1 protein level in cells. In addition, we found that USP10 interacts with RUNX1 through the N-terminal 1–206 fragment of USP10 and the N-terminal sequences containing the Runt domain (amino acids 1–204) of RUNX1. DUBs were known to remove the ubiquitin from ubiquitinated protein and neutralize the function of E3-ubiquitin ligases with a complete pattern [49]. We confirmed that the catalytic activity of USP10 was key to regulate RUNX1 protein stability and GBM malignancy. Lys48- and Lys63-linked chains are two major forms of polyubiquitin chains that associate with the proteasome pathway-mediated protein degradation [23, 41]. Indeed, USP10 significantly degrades the lys48-polyubiquitin chain of RUNX1, but has no effect on the lys63-polyubiquitin chain. These results indicate that USP10 controls RUNX1 protein degradation through the ubiquitin proteasome pathway, independent of the endosomal–lysosomal pathway. Moreover, GBM patients that express high levels of USP10 exhibited poor overall and disease-free survival, while GBM tissue samples confirmed the significant correlation between USP10 and RUNX1. Altogether, our data suggest that the USP10/RUNX1 axis may have predictive value in the prognosis of GBM patients.

## Conclusions

In conclusion, we identified USP10 as a key RUNX1 stabilizing deubiquitinating enzyme that plays a key role in the PMT process of GBM. The USP10/RUNX1 axis can maintain MES properties of GBM cells and promote the development of GBM (Fig. 7F). Our findings contribute to a better understanding of the molecular mechanism underlying PMT in GBM, and reveal that small molecule inhibitors of USP10 may represent a novel strategy for GBM treatment.

## Supplementary information

Supplementary Figure1

Supplementary Figure2

Supplementary Figure3

Supplementary Figure4

Supplementary Figure5

Supplementary Figure6

Supplementary Figure7

Supplementary Table

Supplementary figure and table legends

Original images of western blot

Reproducibility checklist

## Data Availability

The data that support the findings of this study are available from the corresponding author upon reasonable request.
