# Supplementary figures and images for: USP10 deubiquitinates RUNX1 and promotes proneural-to-mesenchymal transition in glioblastoma
Source: Cell Death Dis. 2023 Mar 22;14(3):207. doi: 10.1038/s41419-023-05734-y (PMC10033651; doi:10.1038/s41419-023-05734-y)

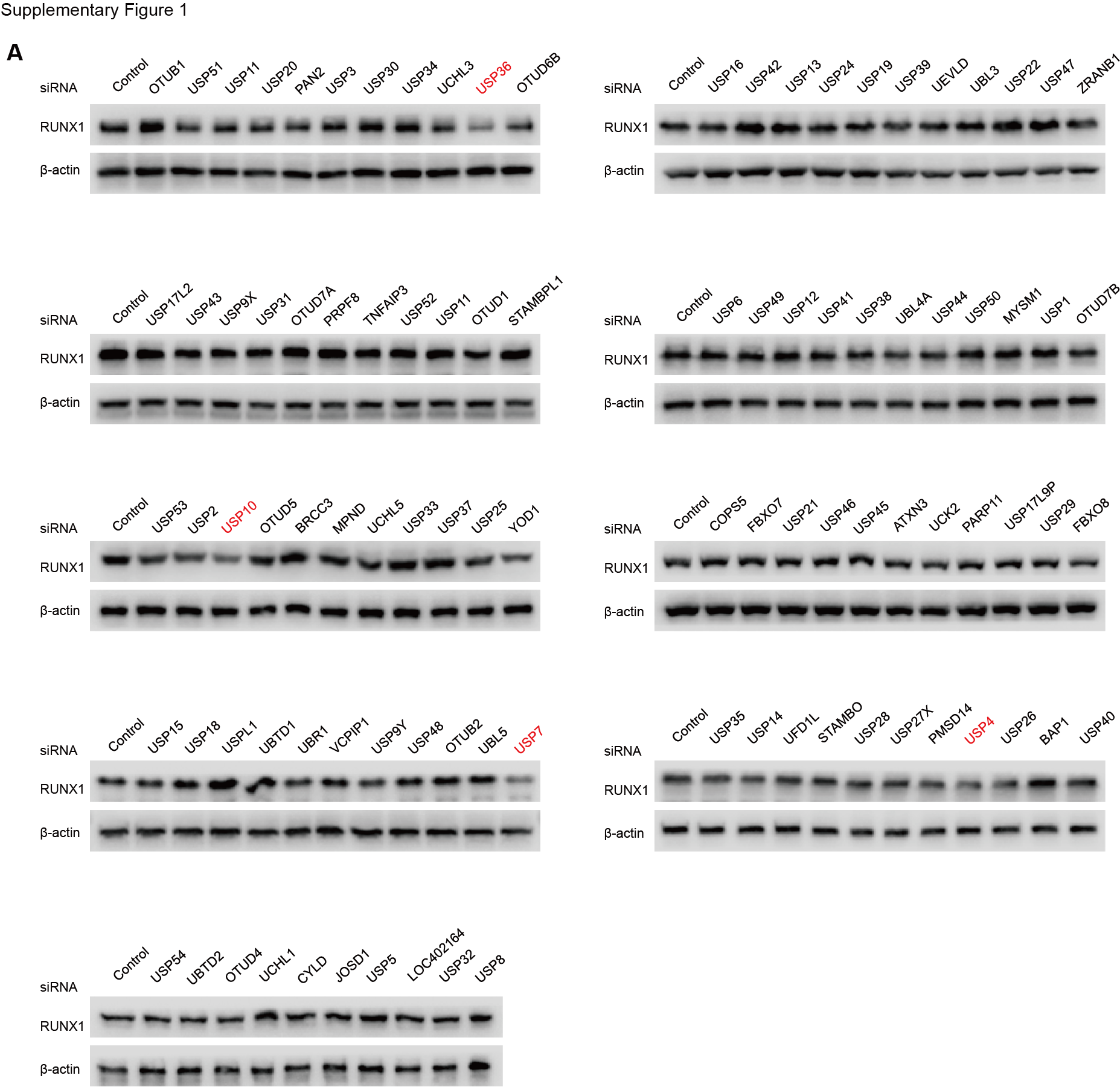

Supplement: Supplementary file 1 — Supplementary Figure1 [file 41419_2023_5734_MOESM1_ESM.tif]

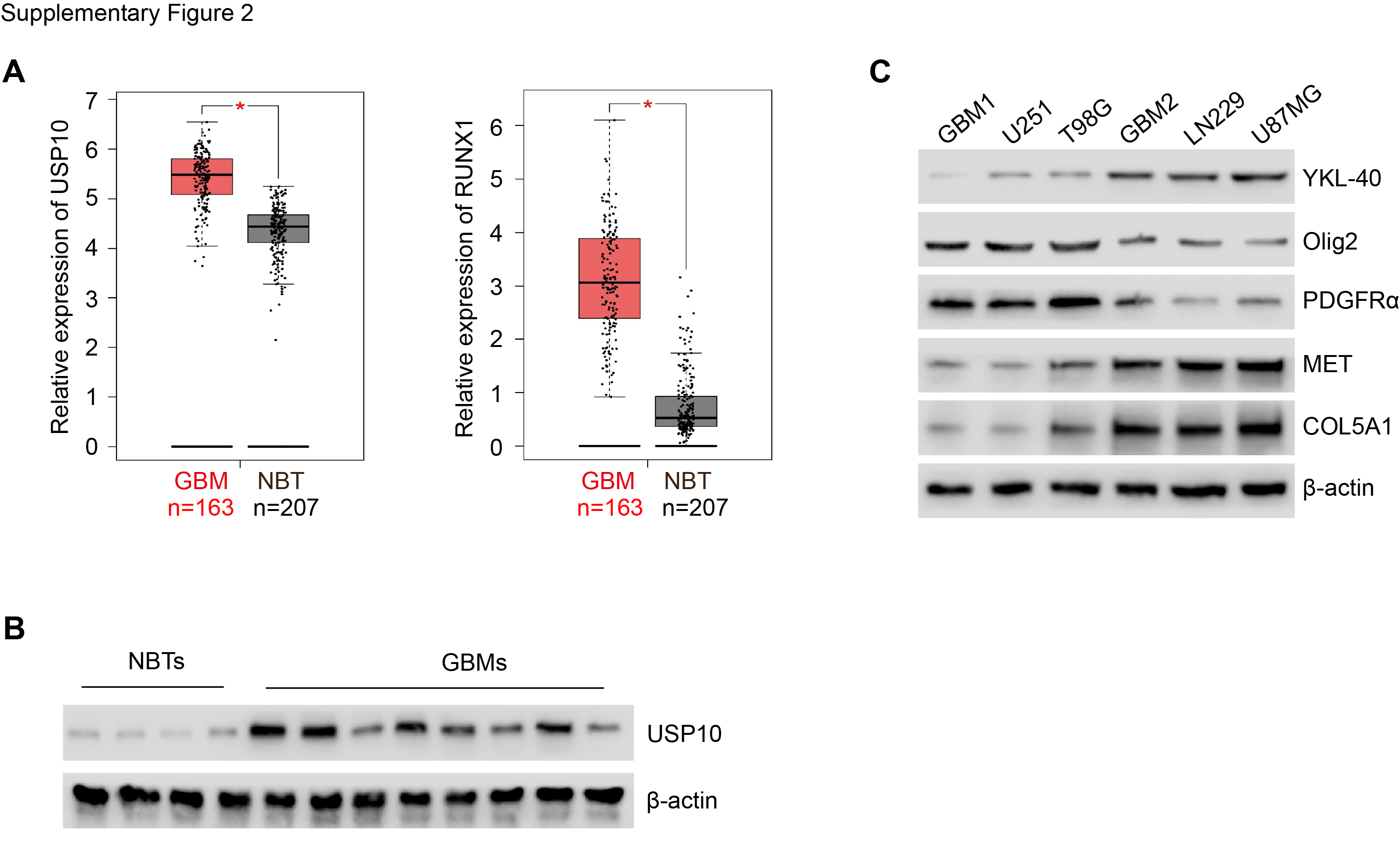

Supplement: Supplementary file 2 — Supplementary Figure2 [file 41419_2023_5734_MOESM2_ESM.tif]

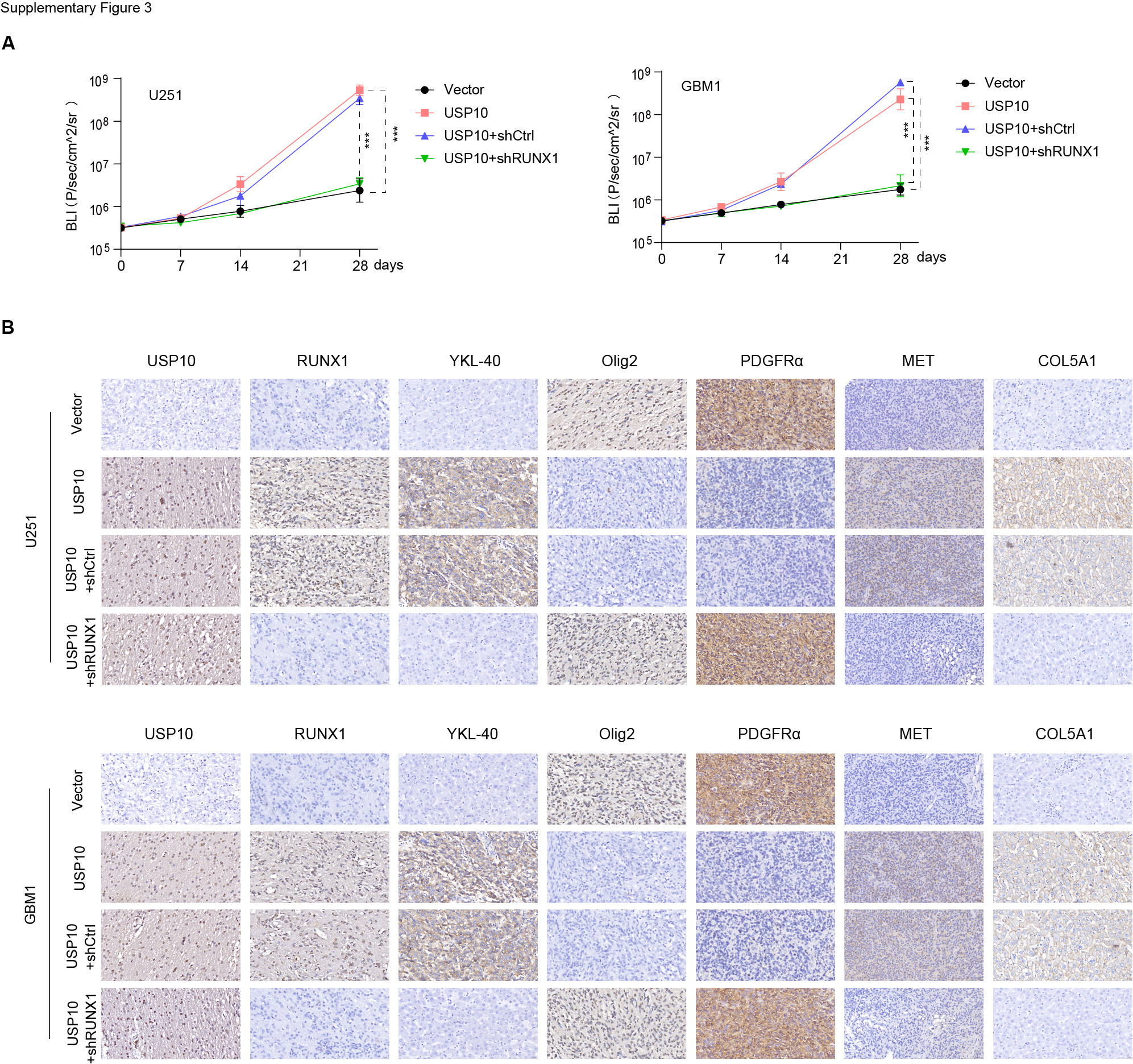

Supplement: Supplementary file 3 — Supplementary Figure3 [file 41419_2023_5734_MOESM3_ESM.tif]

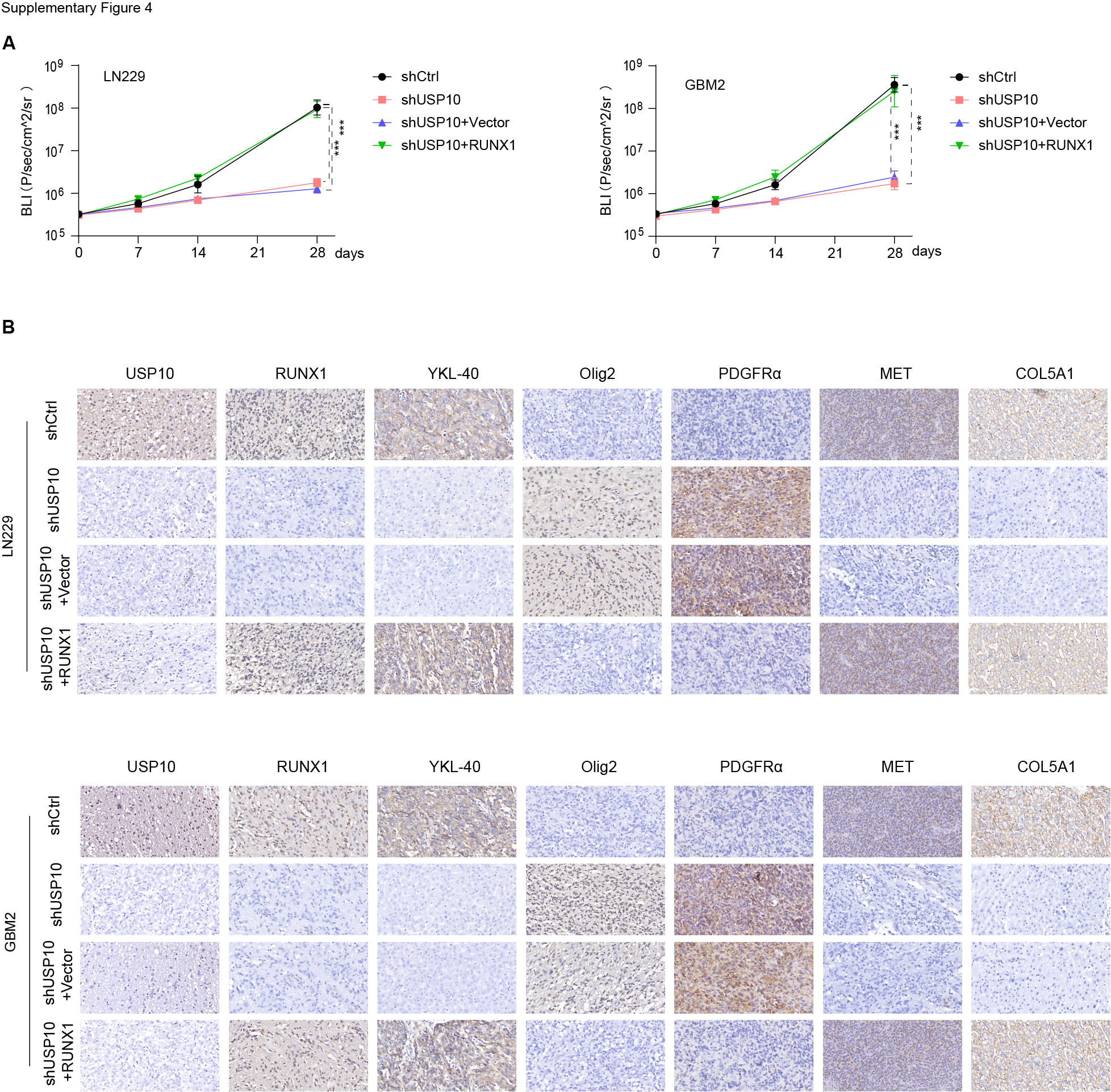

Supplement: Supplementary file 4 — Supplementary Figure4 [file 41419_2023_5734_MOESM4_ESM.tif]

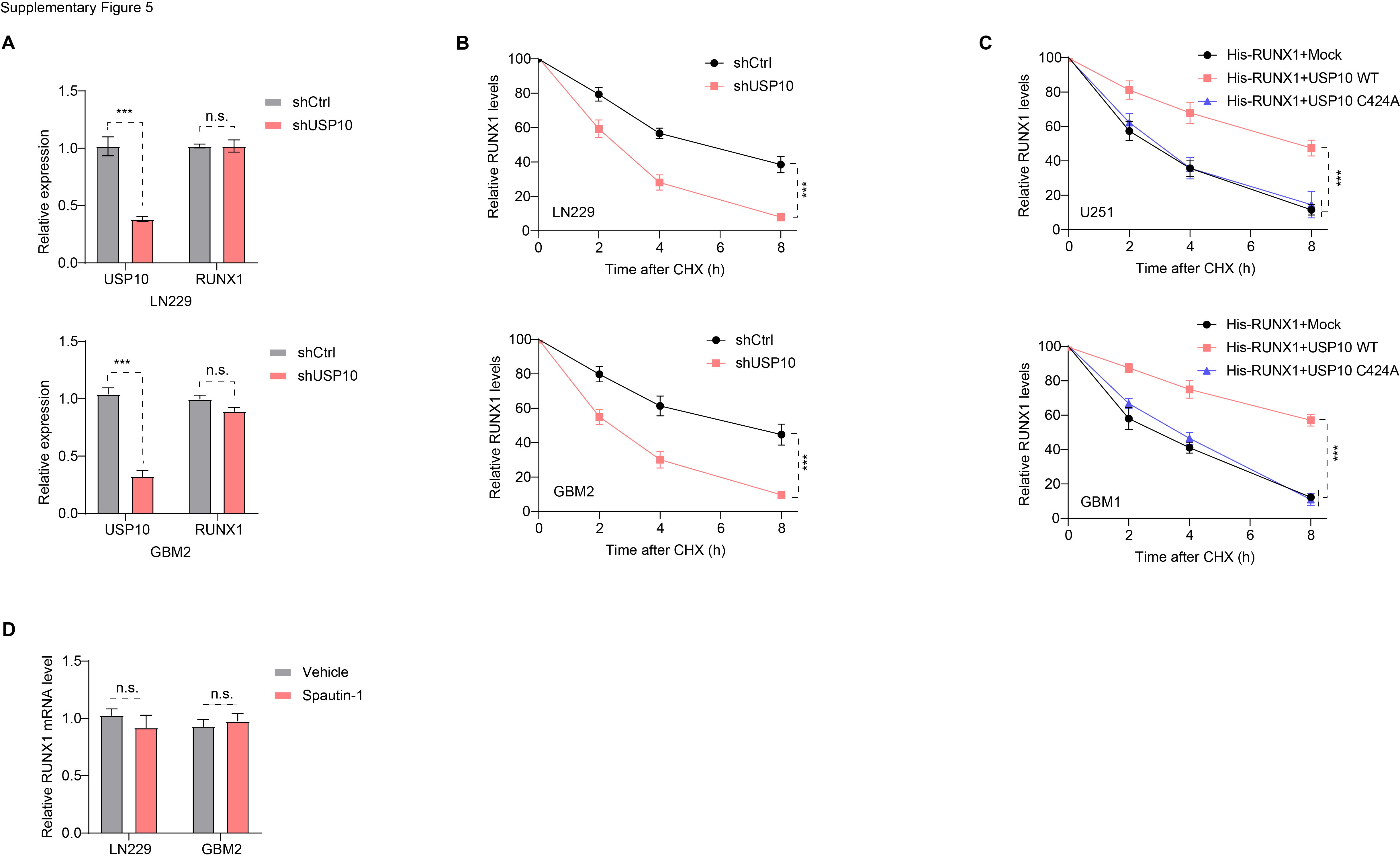

Supplement: Supplementary file 5 — Supplementary Figure5 [file 41419_2023_5734_MOESM5_ESM.tif]

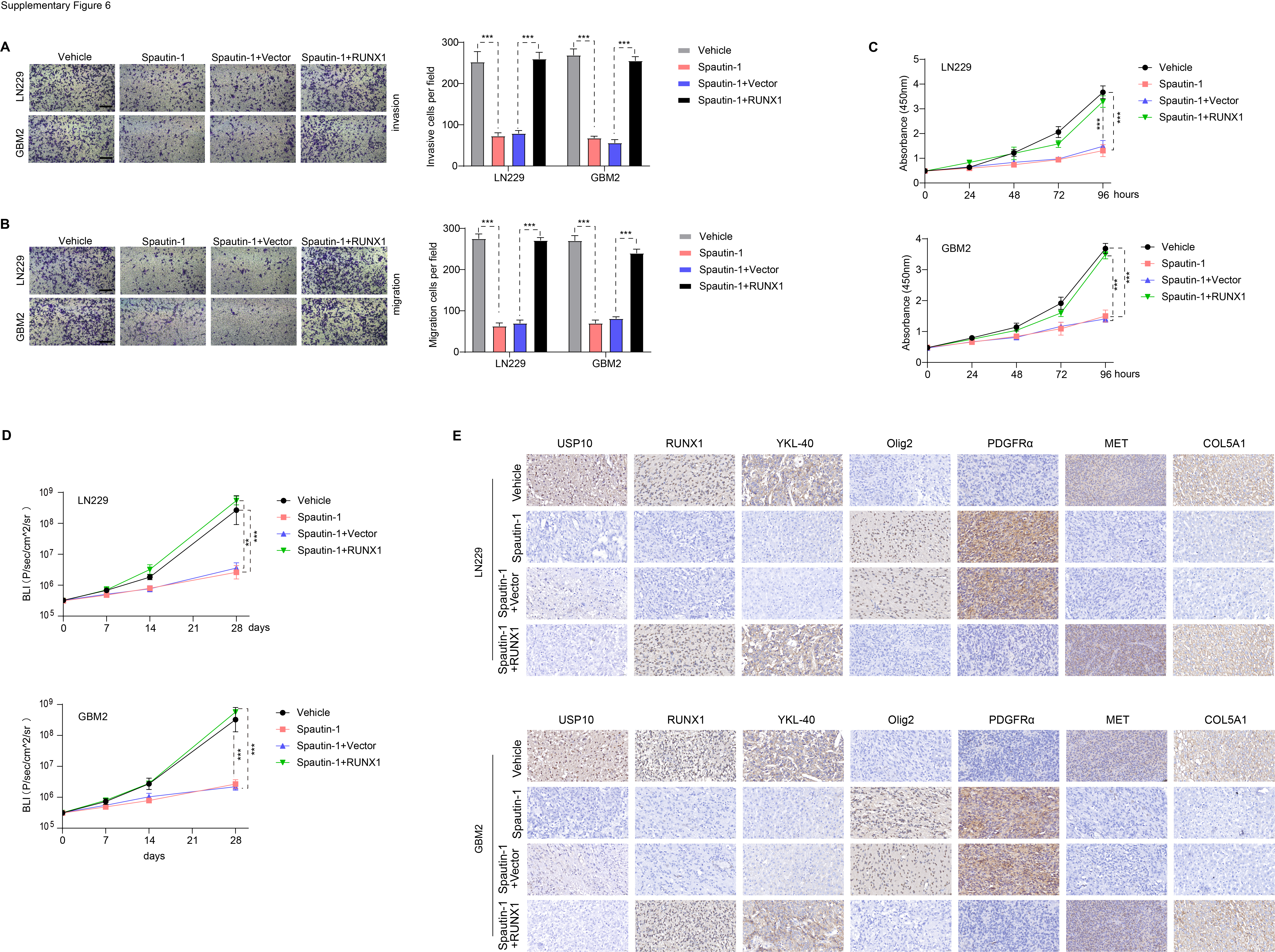

Supplement: Supplementary file 6 — Supplementary Figure6 [file 41419_2023_5734_MOESM6_ESM.tif]

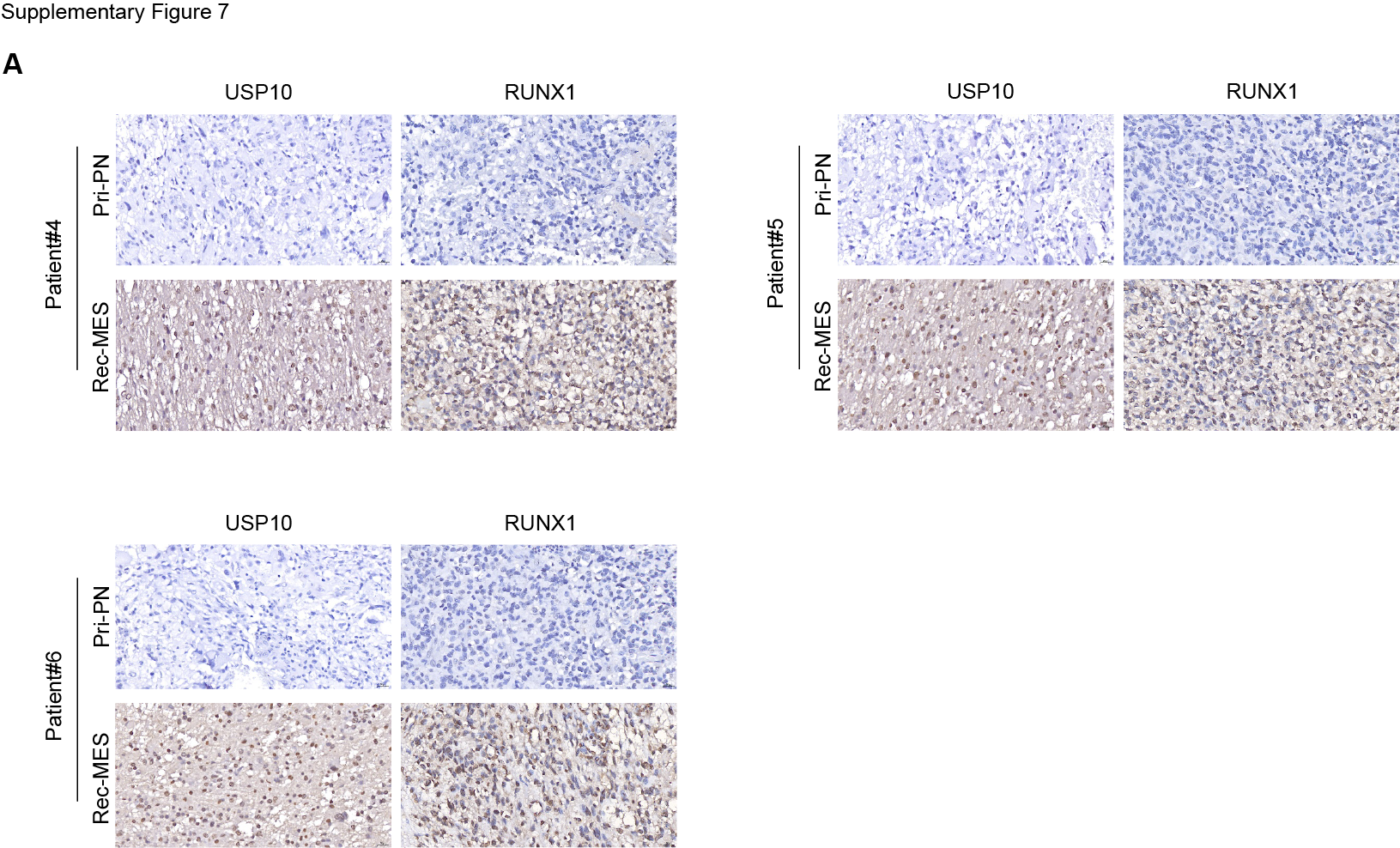

Supplement: Supplementary file 7 — Supplementary Figure7 [file 41419_2023_5734_MOESM7_ESM.tif]
