# Supplementary material for: USP10 deubiquitinates RUNX1 and promotes proneural-to-mesenchymal transition in glioblastoma
Source: Cell Death Dis. 2023 Mar 22;14(3):207. doi: 10.1038/s41419-023-05734-y (PMC10033651; doi:10.1038/s41419-023-05734-y)
Supplement: Supplementary file 8 — Supplementary Table [file 41419_2023_5734_MOESM8_ESM.docx]

**Supplementary Table S1. 58 GBM patients clinicopathological characteristic**


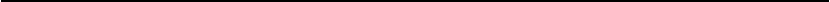


Characteristic Value


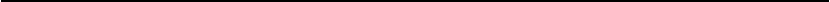


| Total samples (n) | 58 |
| --- | --- |
| Sex (n) |  |
| Male | 28 |
| Female | 30 |
| Medium age, years (range) | 51 (21-74) |
| Tumor location |  |
| Frontal | 31 |
| Non-frontal | 27 |
| Medium KPS (range) | 80 (40-90) |


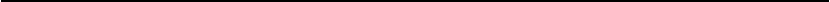


Abbreviations: KPS, Karnofsky performance status; WHO, World Health Organization.
