# Supplementary material for: USP10 deubiquitinates RUNX1 and promotes proneural-to-mesenchymal transition in glioblastoma
Source: Cell Death Dis. 2023 Mar 22;14(3):207. doi: 10.1038/s41419-023-05734-y (PMC10033651; doi:10.1038/s41419-023-05734-y)
Supplement: Supplementary file 9 — Supplementary figure and table legends [file 41419_2023_5734_MOESM9_ESM.docx]

**Supplementary figure and table legends**

**Fig. S1.** **A** DUB siRNA library screening revealed that siRNA-mediated inhibition of multiple DUB genes decreased RUNX1 protein levels.

**Fig. S2. A** USP10 and RUNX1 expression analyzed using the GEPIA database. **B** Western blot showing USP10 protein levels in normal brain tissues and GBM tissues. **C** Western blot showing the PN subtype markers (Olig2, PDGFRα) and MES subtype markers (YKL-40, MET, and COL5A1) in four established and two primary GBM cell lines. **p < 0.01.

**Fig. S3. A** Bioluminescence was quantified in U251- and GBM1-derived tumors from four groups. **B** Immunohistochemical staining of orthotopic xenograft tissues showing expression of USP10, RUNX1, PN subtype markers (Olig2, PDGFRα) and MES subtype markers (YKL-40, MET, and COL5A1) with indicated modifications expressing control vector /USP10 or shCtrl /shRUNX1. ***p < 0.001.

**Fig. S4. A** Bioluminescence was quantified in LN229- and GBM2-derived tumors from four groups. **B** Immunohistochemical staining of orthotopic xenograft tissues showing expression of USP10, RUNX1, PN subtype markers (Olig2, PDGFRα) and MES subtype markers (YKL-40, MET, and COL5A1) with indicated modifications transduced with shCtrl/shUSP10 or control vector /RUNX1. ***p < 0.001.

**Fig. S5. A** RUNX1 mRNA levels in LN229 and GBM2 cells with USP10 knockdown. **B** Quantifications of RUNX1 expression in LN229/GBM2 cells normalized to β-actin. **C** Quantifications of RUNX1 expression in U251/GBM1 cells normalized to β-actin. **D** RUNX1 mRNA levels in LN229 and GBM2 cells with or without Spautin-1 (1 μM). ***p < 0.001; n.s., not significant.

**Fig. S6. A** and **B** Invasion (A) and migration (B) of indicated cells measured using the Transwell assay. Scale bar: 100 μm. Right panel shows quantification results. **C** Representative bar graph showing the proliferation of indicated cells with different treatments. **D** Bioluminescence was quantified in tumors from four groups. **E** Immunohistochemical staining of orthotopic xenograft tissues showing expression of USP10, RUNX1, PN subtype markers (Olig2, PDGFRα) and MES subtype markers (YKL-40, MET, and COL5A1) derived from LN229 and GBM2 cells treated with vehicle or 20 mg/kg Spautin-1, reconstituted with vector control or RUNX1. **p < 0.01; ***p < 0.001.

**Fig. S7.** **A** USP10 and RUNX1 expression in two matched pairs of relapsed MES and primary PN tumors.

**Supplementary Table S1.** Clinicopathological characteristics of 58 GBM patients**.**
