# Supplementary material for: USP10 deubiquitinates RUNX1 and promotes proneural-to-mesenchymal transition in glioblastoma
Source: Cell Death Dis. 2023 Mar 22;14(3):207. doi: 10.1038/s41419-023-05734-y (PMC10033651; doi:10.1038/s41419-023-05734-y)

Figure 1B

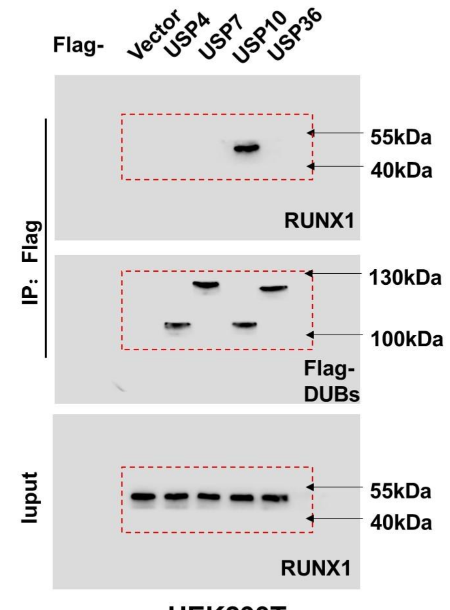

HEK293T

Figure 1C

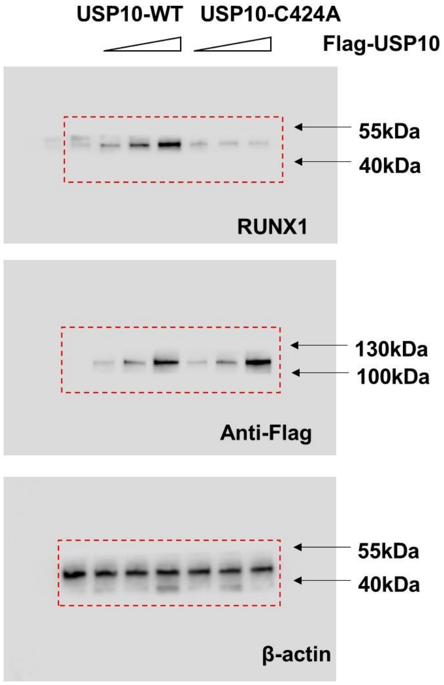

HEK293T

Figure 1F

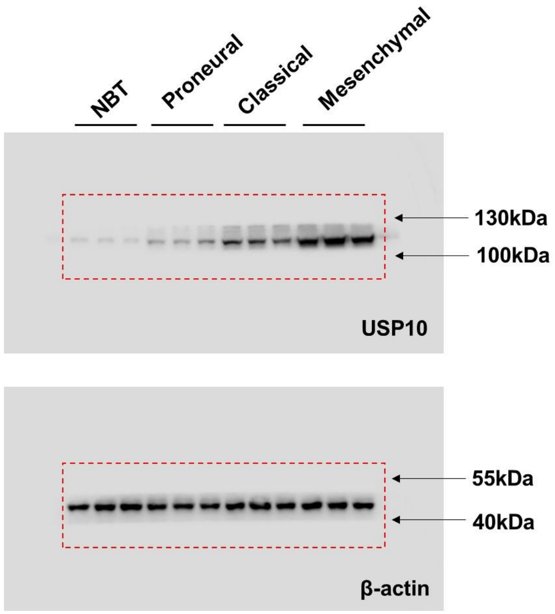

Figure 1G

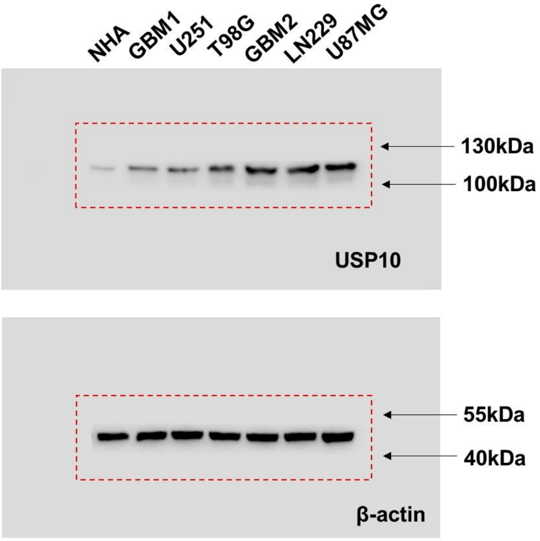

Figure 1H

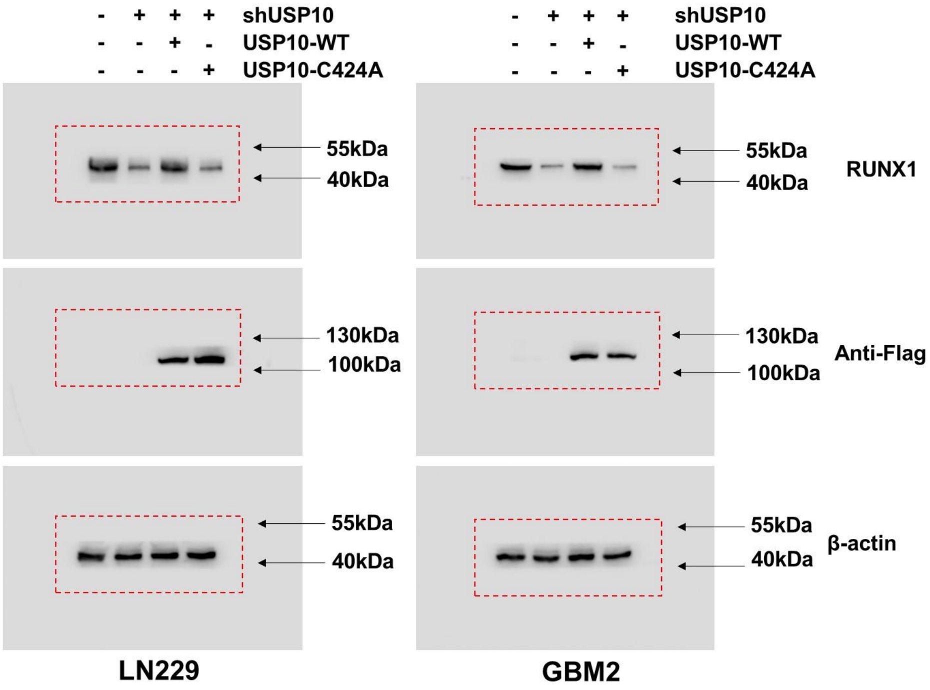

Figure 2A

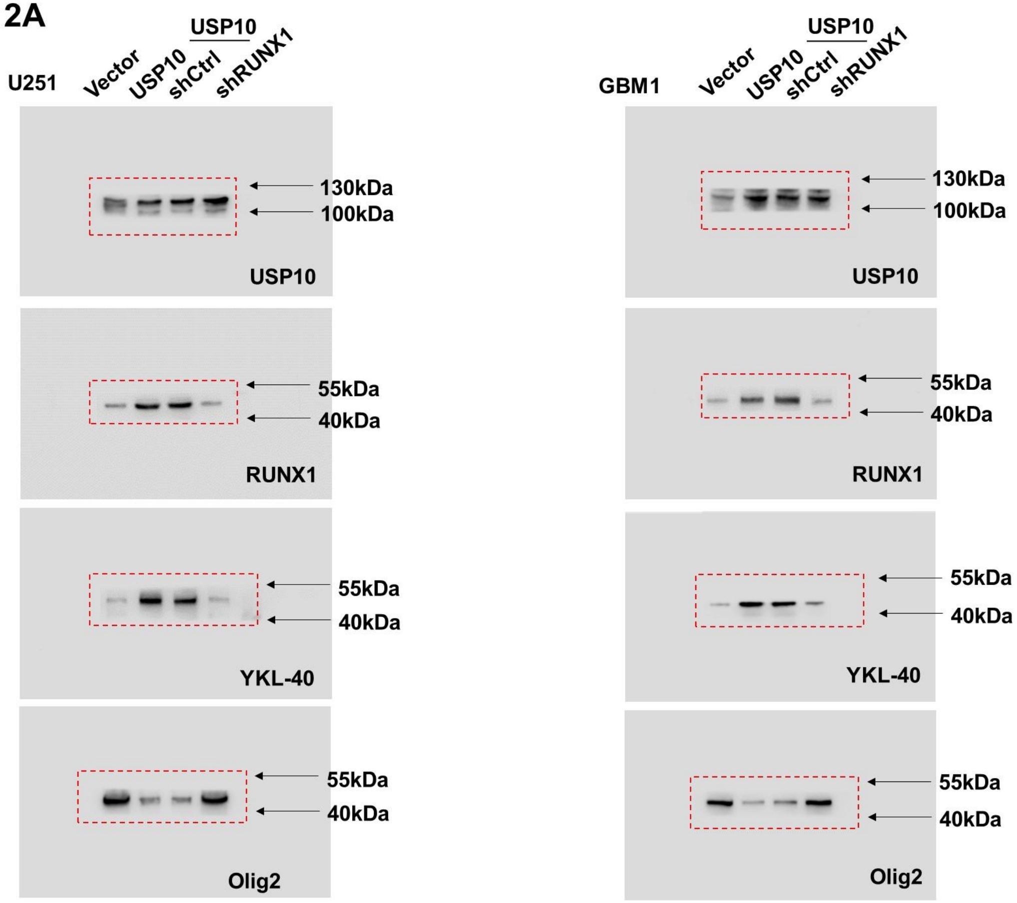
U251
Vector
USP10
USP10
shCtrl
shRUNX1
130kDa
100kDa
USP10
55kDa
40kDa
RUNX1
55kDa
40kDa
YKL-40
55kDa
40kDa
Olig2
GBM1
Vector
USP10
USP10
shCtrl
shRUNX1
130kDa
100kDa
USP10
55kDa
40kDa
RUNX1
55kDa
40kDa
YKL-40
55kDa
40kDa
Olig2

Figure 2A

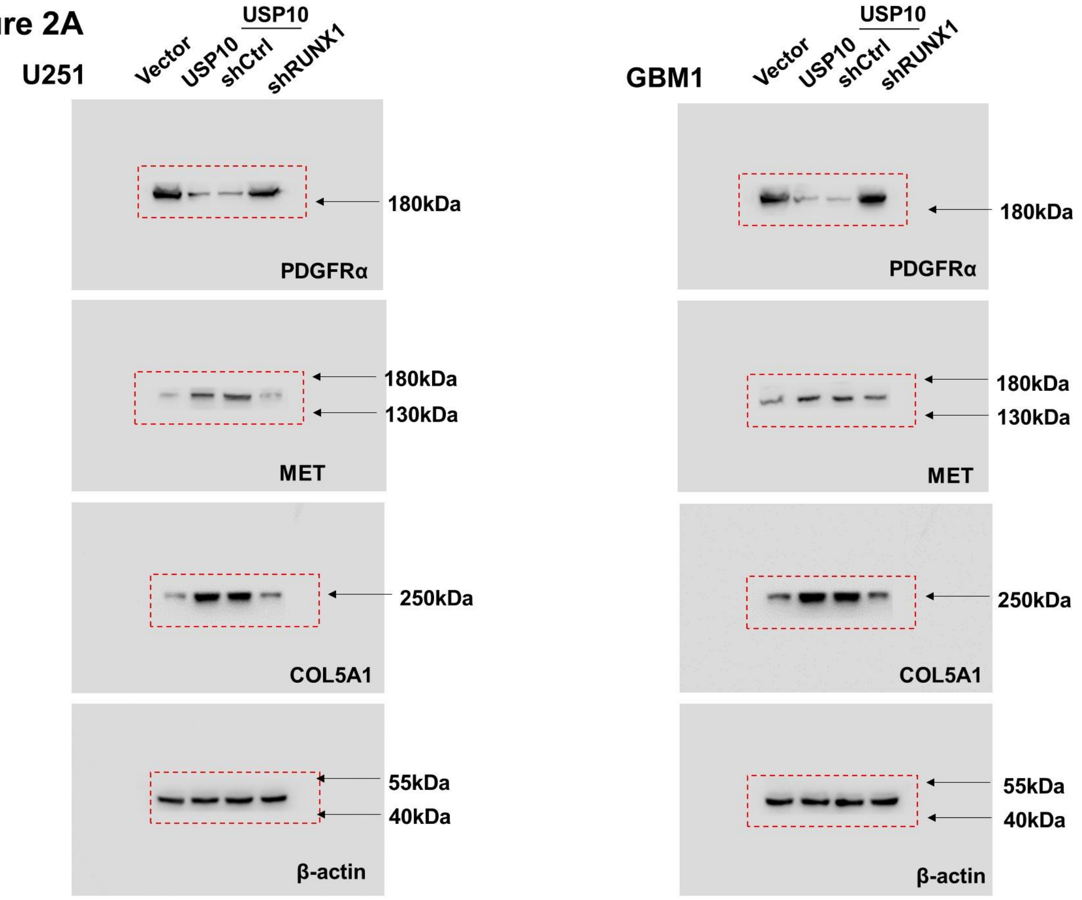
U251
USP10
Vector
USP10
shCtrl
shRUNX1
180kDa
PDGFRα
180kDa
130kDa
MET
250kDa
COL5A1
55kDa
40kDa
β-actin
GBM1
USP10
Vector
USP10
shCtrl
shRUNX1
180kDa
PDGFRα
180kDa
130kDa
MET
250kDa
COL5A1
55kDa
40kDa
β-actin

Figure 3A

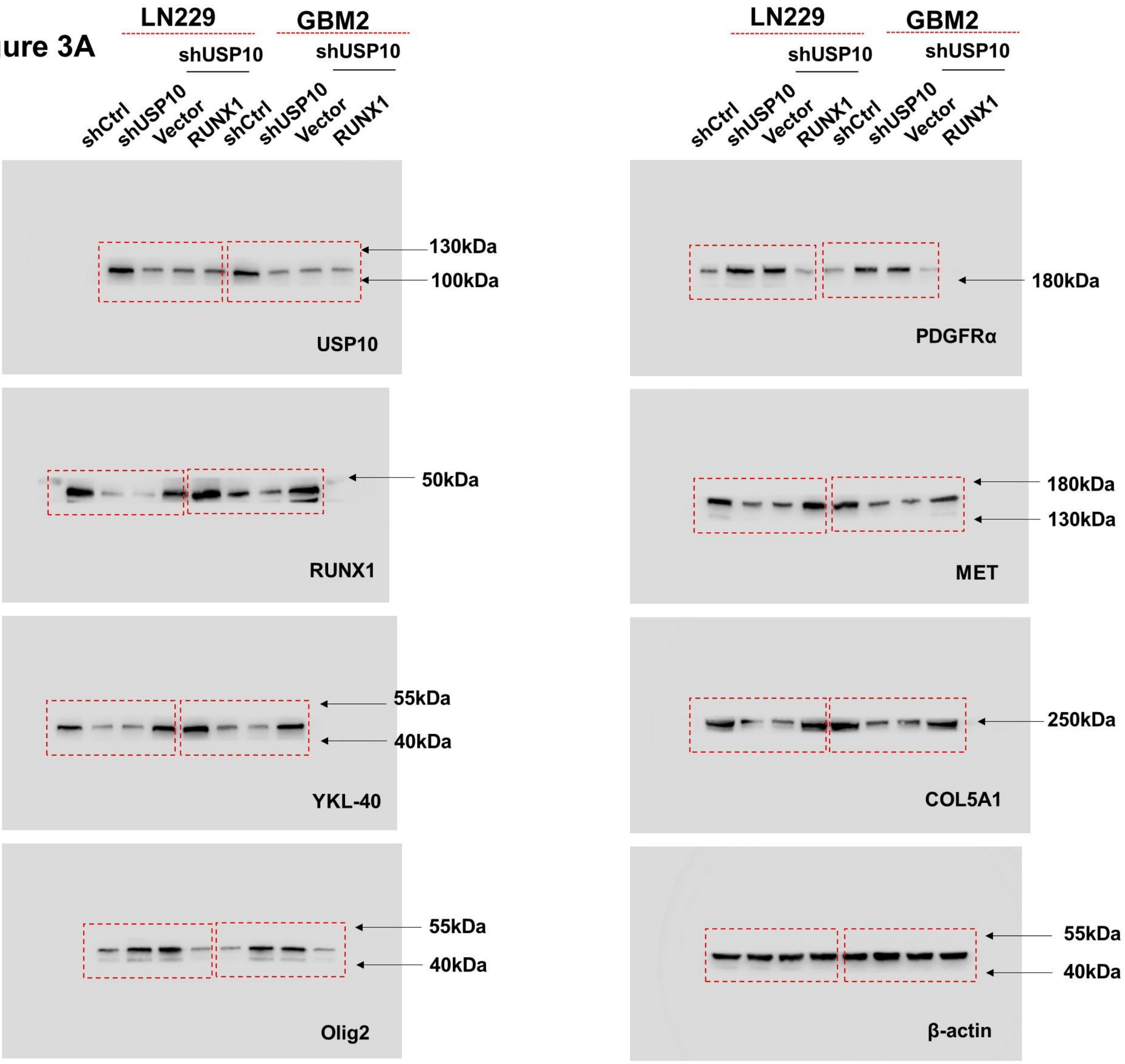
LN229
GBM2
shUSP10
shUSP10
shCtrl
shUSP10
Vector
RUNX1
shCtrl
shUSP10
Vector
RUNX1
130kDa
100kDa
USP10
50kDa
RUNX1
55kDa
40kDa
YKL-40
55kDa
40kDa
Olig2
LN229
GBM2
shUSP10
shUSP10
shCtrl
shUSP10
Vector
RUNX1
shCtrl
shUSP10
Vector
RUNX1
180kDa
PDGFRα
180kDa
130kDa
MET
250kDa
COL5A1
55kDa
40kDa
β-actin

Figure 4A

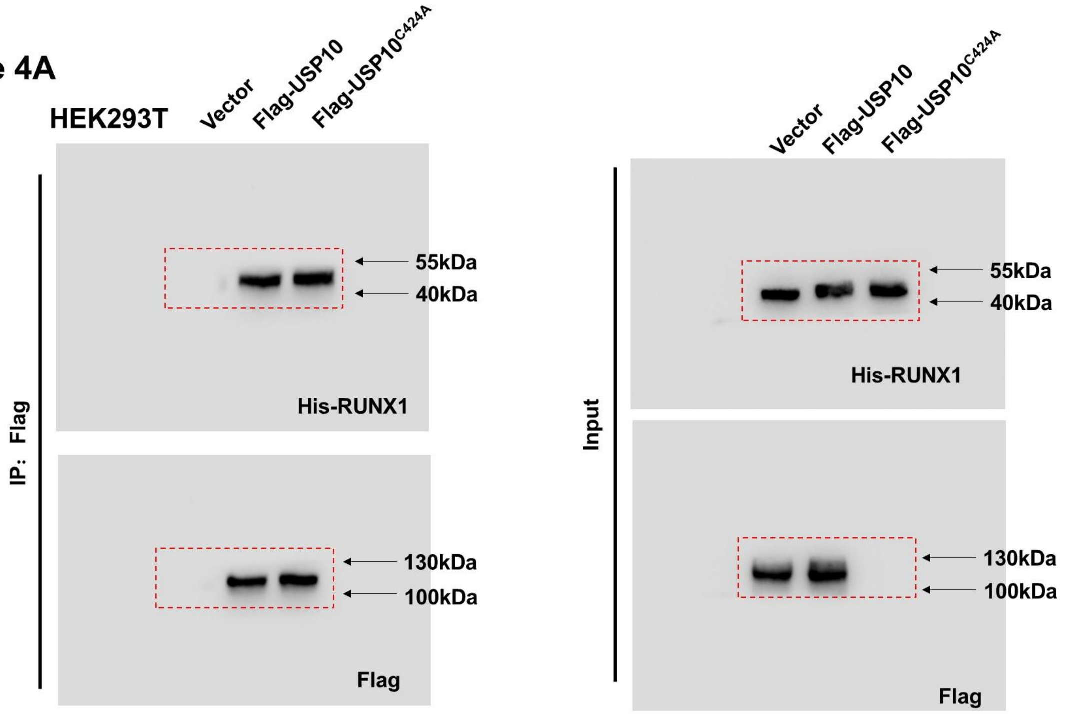

Figure 4B

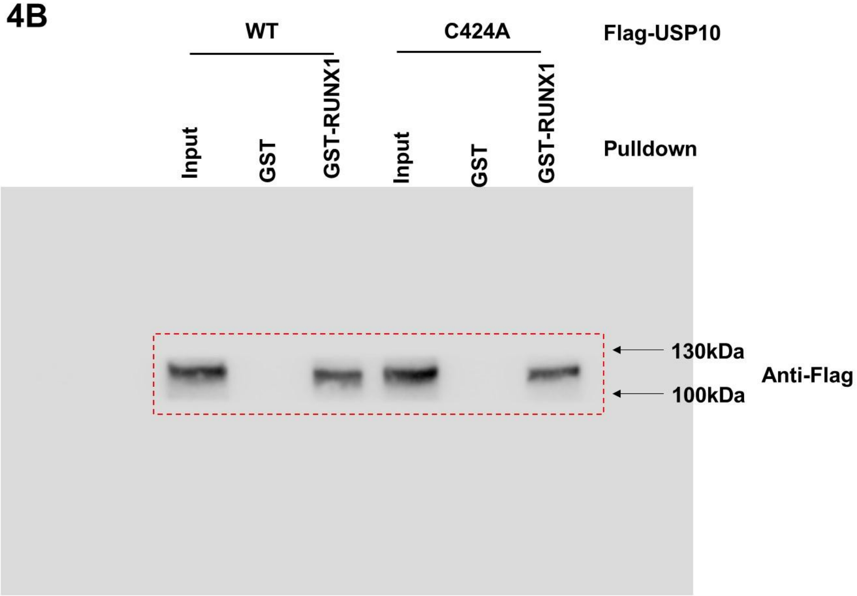

Figure 4D

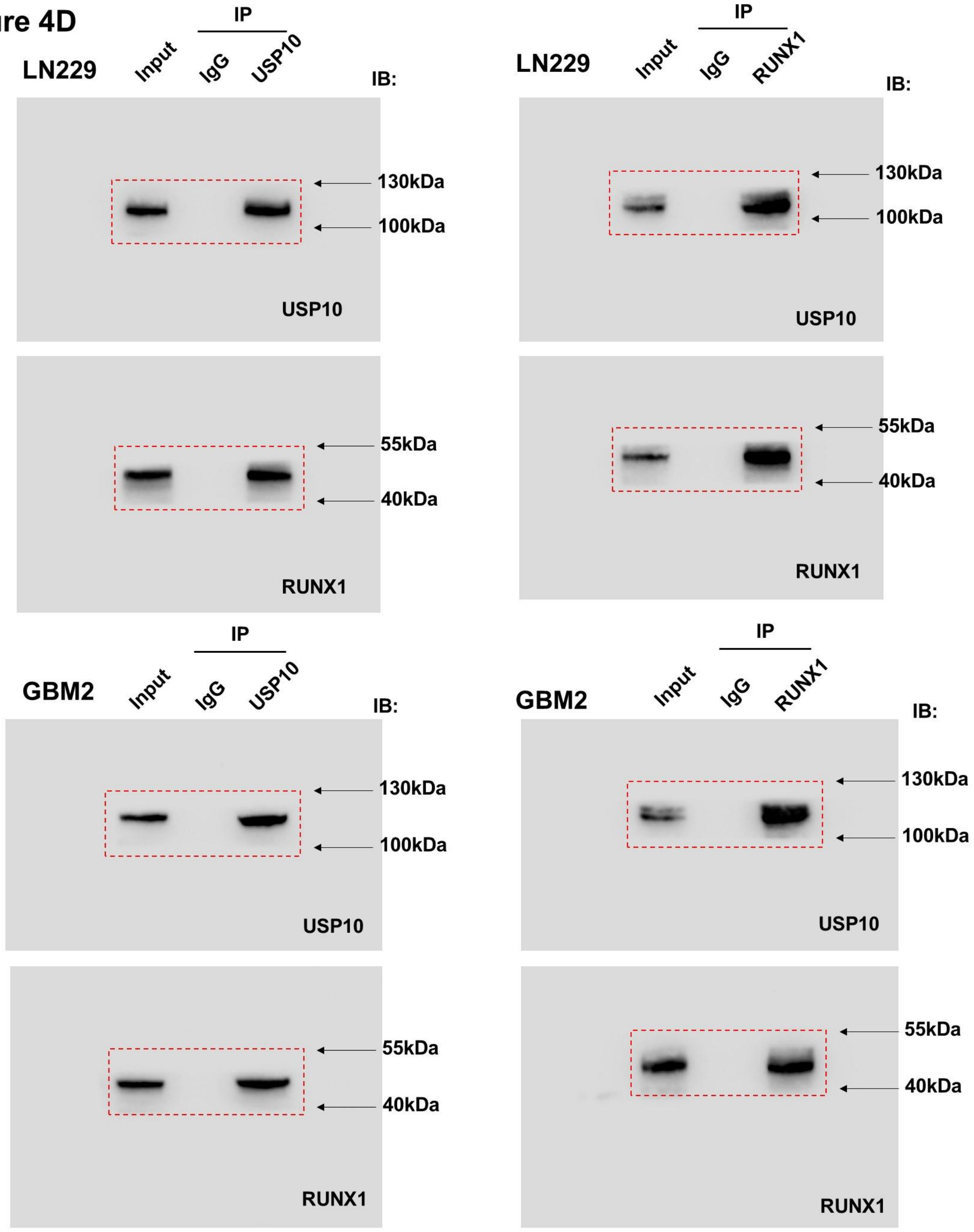
LN229
Input
IgG
USP10
IP
IB:
130kDa
100kDa
USP10
55kDa
40kDa
RUNX1
LN229
Input
IgG
RUNX1
IP
IB:
130kDa
100kDa
USP10
55kDa
40kDa
RUNX1
GBM2
Input
IgG
USP10
IP
IB:
130kDa
100kDa
USP10
55kDa
40kDa
RUNX1
GBM2
Input
IgG
RUNX1
IP
IB:
130kDa
100kDa
USP10
55kDa
40kDa
RUNX1

Figure 4F

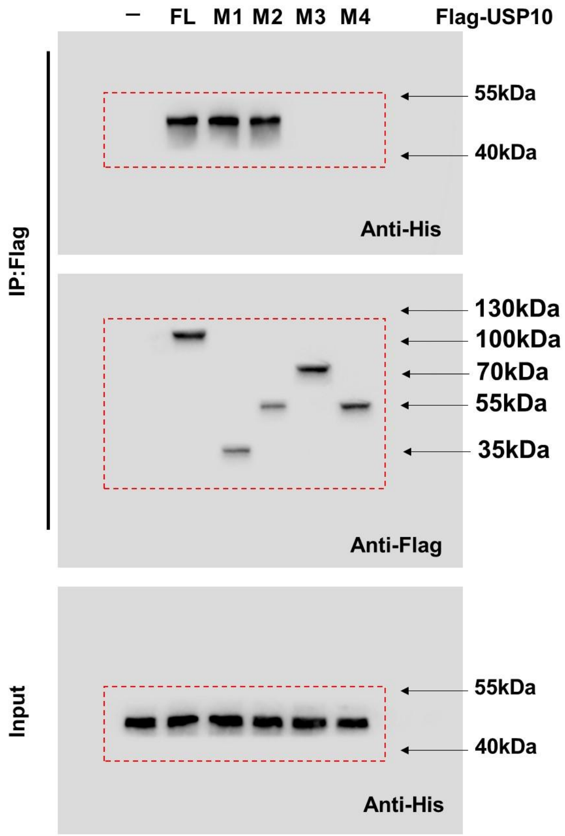

Figure 4G

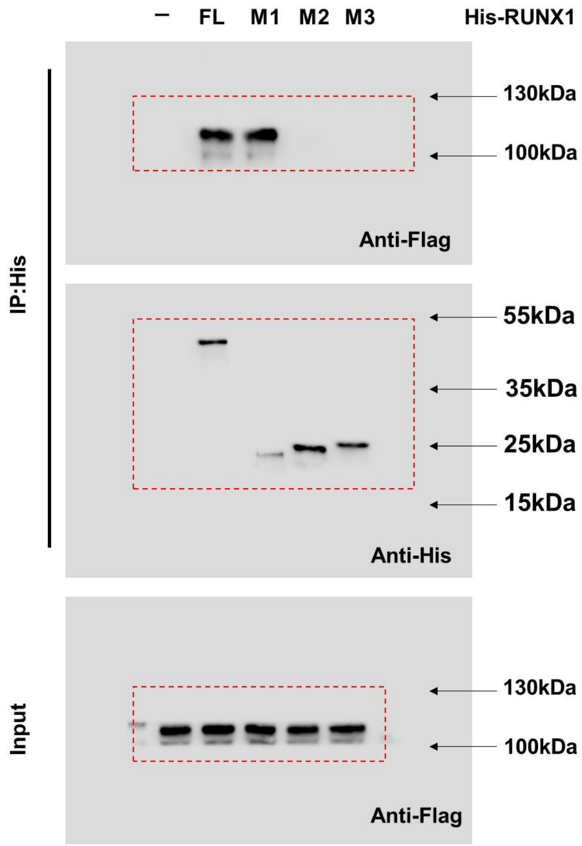

Figure 5A

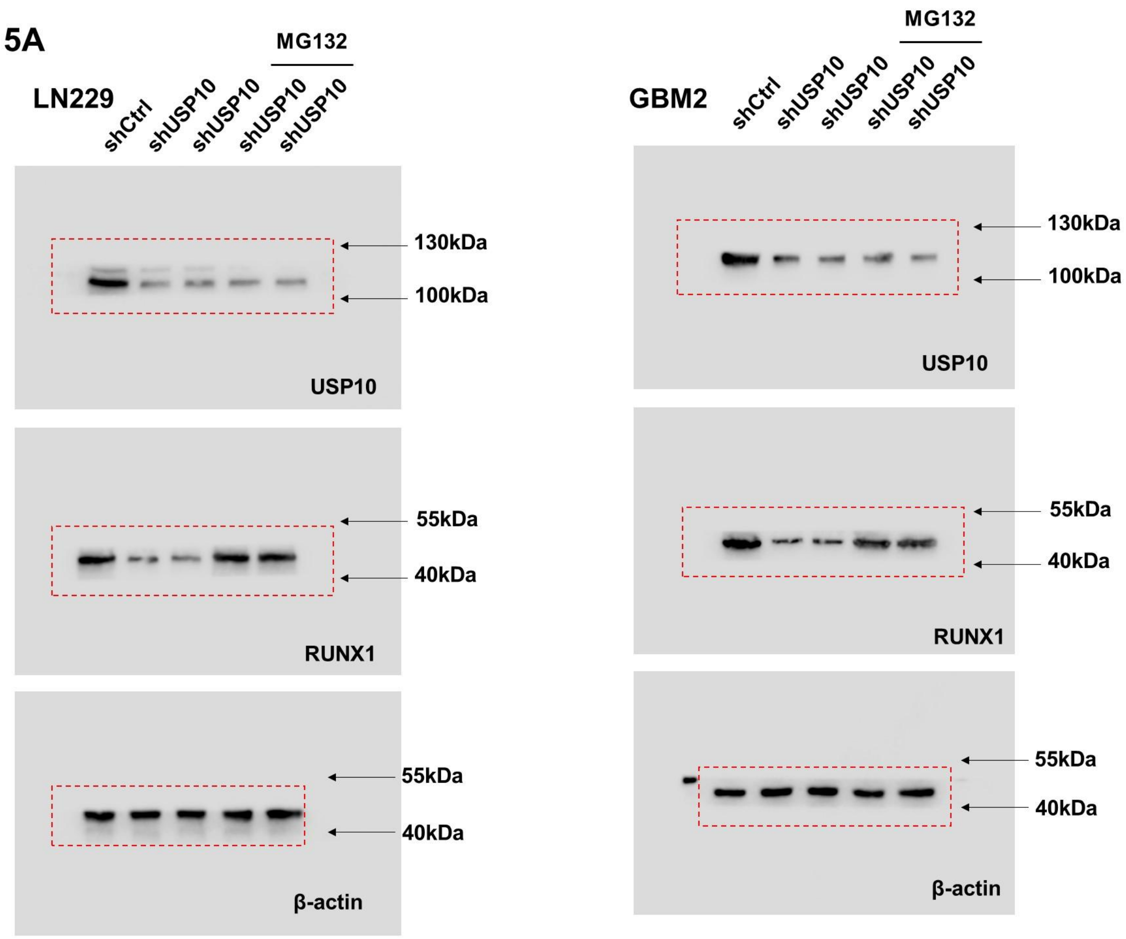
LN229
MG132
shCtrl
shUSP10
shUSP10
shUSP10
shUSP10
130kDa
100kDa
USP10
55kDa
40kDa
RUNX1
55kDa
40kDa
β-actin
GBM2
MG132
shCtrl
shUSP10
shUSP10
shUSP10
shUSP10
130kDa
100kDa
USP10
55kDa
40kDa
RUNX1
55kDa
40kDa
β-actin

Figure 5B

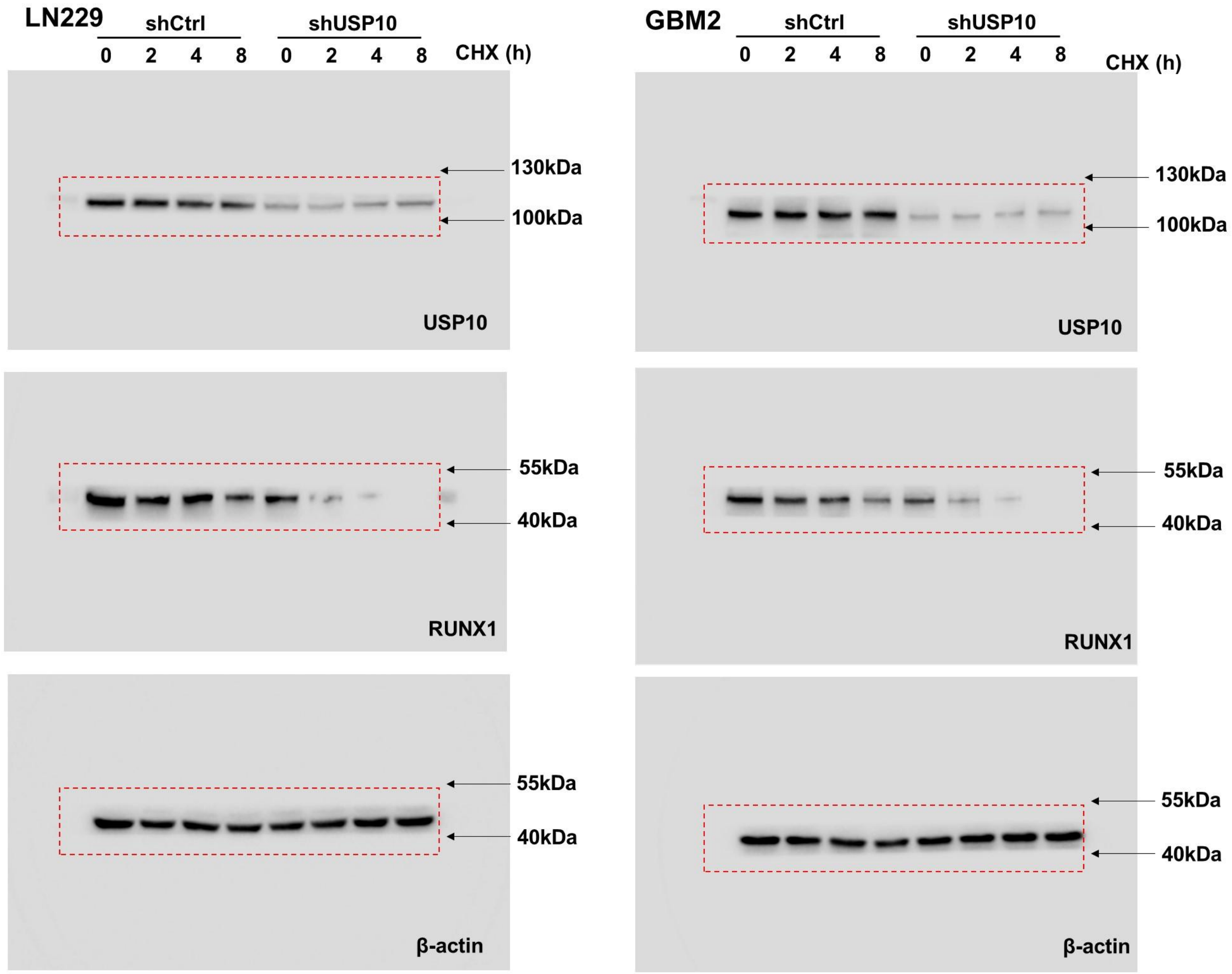
LN229
shCtrl
shUSP10
0 2 4 8 0 2 4 8 CHX (h)
130kDa
100kDa
USP10
55kDa
40kDa
RUNX1
55kDa
40kDa
β-actin
GBM2
shCtrl
shUSP10
0 2 4 8 0 2 4 8 CHX (h)
130kDa
100kDa
USP10
55kDa
40kDa
RUNX1
55kDa
40kDa
β-actin

Figure 5C

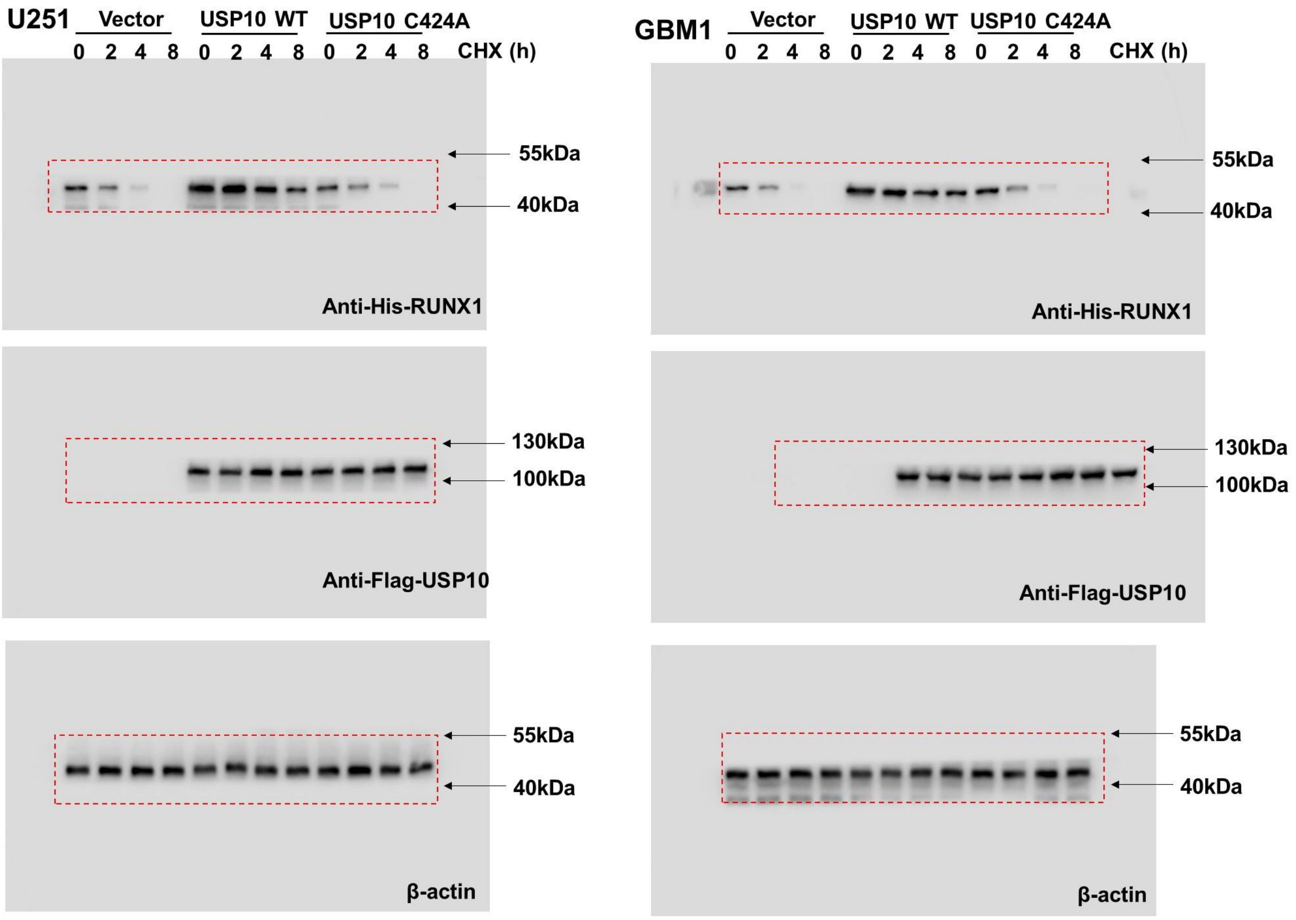
U251
Vector
USP10 WT
USP10 C424A
0 2 4 8 0 2 4 8 0 2 4 8 CHX (h)
55kDa
40kDa
Anti-His-RUNX1
130kDa
100kDa
Anti-Flag-USP10
55kDa
40kDa
β-actin
GBM1
Vector
USP10 WT
USP10 C424A
0 2 4 8 0 2 4 8 0 2 4 8 CHX (h)
55kDa
40kDa
Anti-His-RUNX1
130kDa
100kDa
Anti-Flag-USP10
55kDa
40kDa
β-actin

Figure 5D

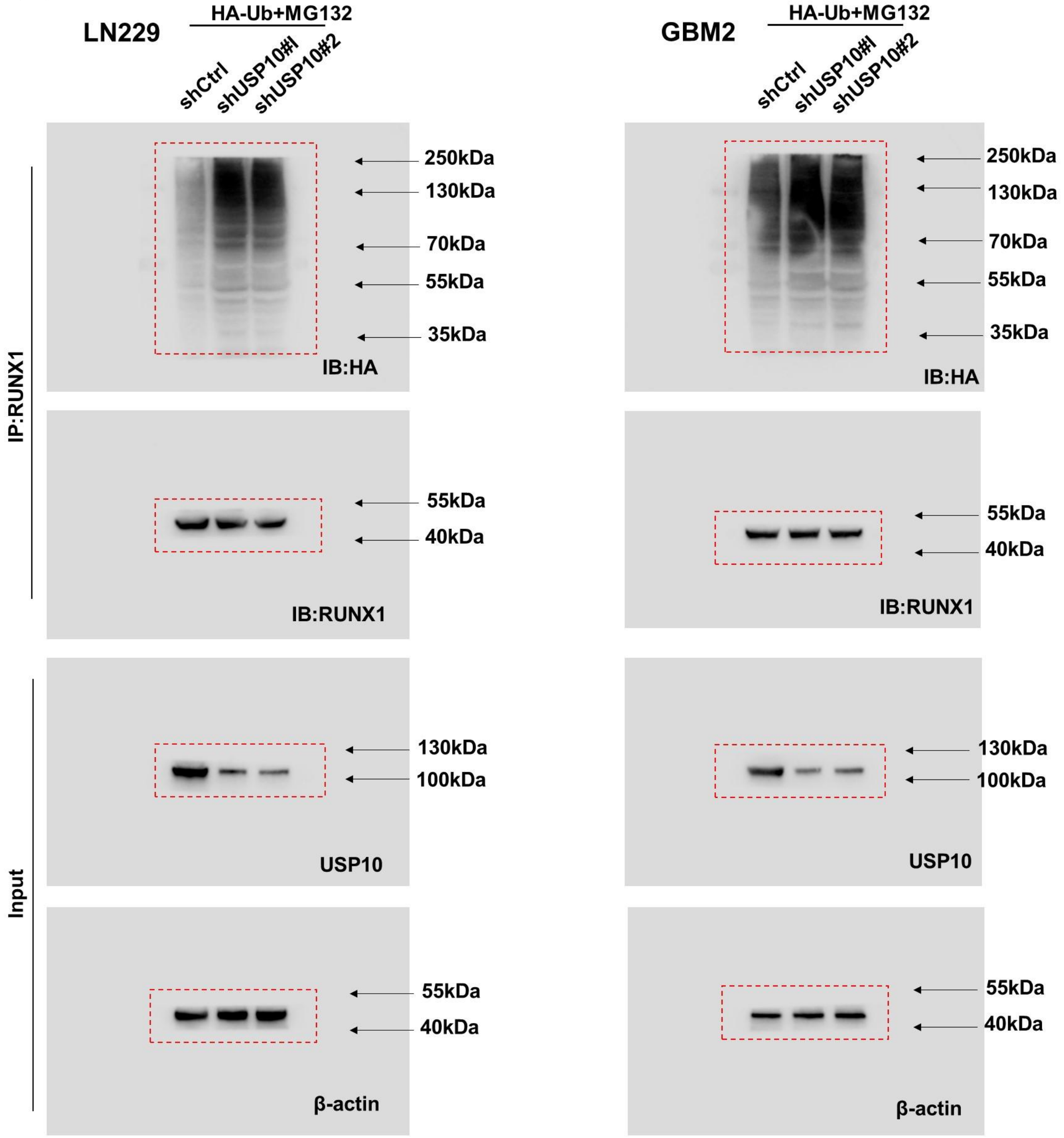
LN229
HA-Ub+MG132
shCtrl
shUSP10#l
shUSP10#2
GBM2
HA-Ub+MG132
shCtrl
shUSP10#l
shUSP10#2
IP:RUNX1
250kDa
130kDa
70kDa
55kDa
35kDa
IB:HA
55kDa
40kDa
IB:RUNX1
Input
130kDa
100kDa
USP10
55kDa
40kDa
β-actin

Figure 5E

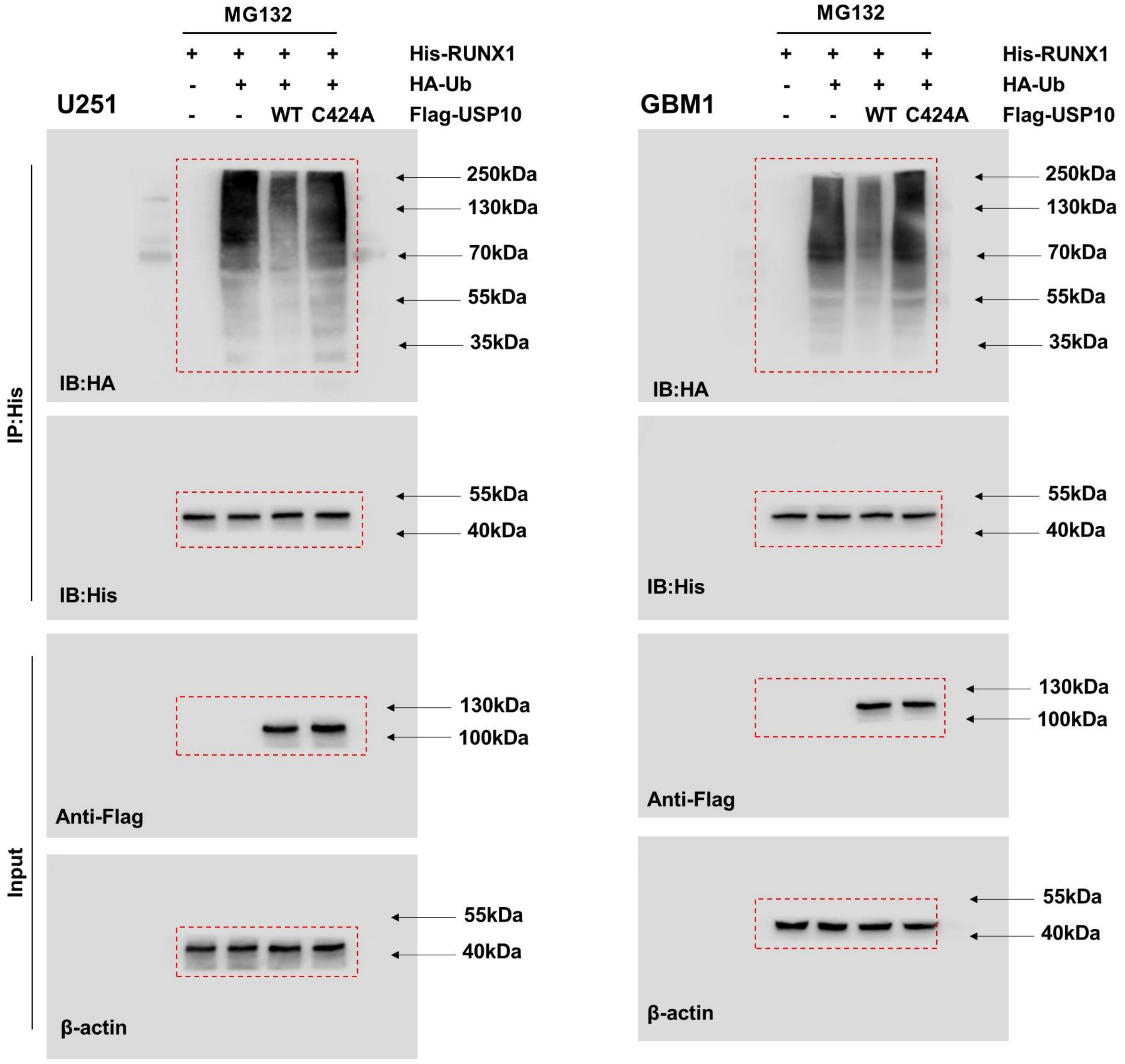
U251
MG132
+ + + + His-RUNX1
- + + + HA-Ub
- - WT C424A Flag-USP10
IP:His
IB:HA
250kDa
130kDa
70kDa
55kDa
35kDa
IB:His
55kDa
40kDa
Input
Anti-Flag
130kDa
100kDa
β-actin
55kDa
40kDa
GBM1
MG132
+ + + + His-RUNX1
- + + + HA-Ub
- - WT C424A Flag-USP10
IB:HA
250kDa
130kDa
70kDa
55kDa
35kDa
IB:His
55kDa
40kDa
Anti-Flag
130kDa
100kDa
β-actin
55kDa
40kDa

Figure 5F

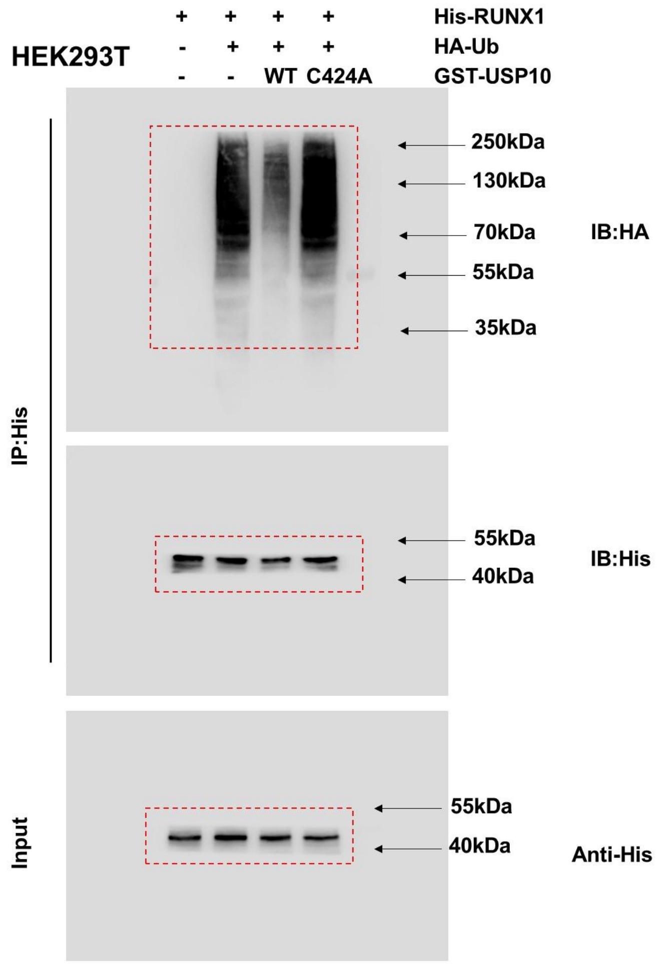
HEK293T
+ + + + His-RUNX1
- + + + HA-Ub
- - WT C424A GST-USP10
IP:His
250kDa
130kDa
70kDa
55kDa
35kDa
IB:HA
55kDa
40kDa
IB:His
Input
55kDa
40kDa
Anti-His

Figure 5G

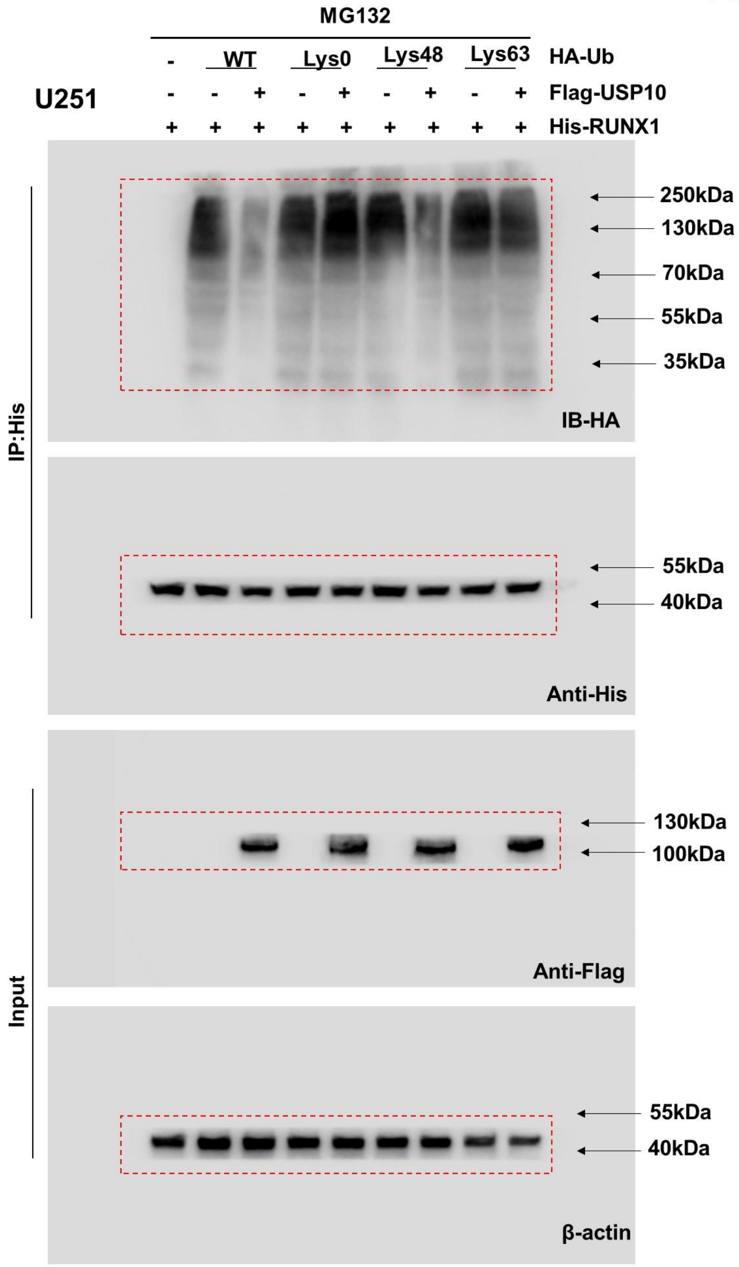

Figure 5H

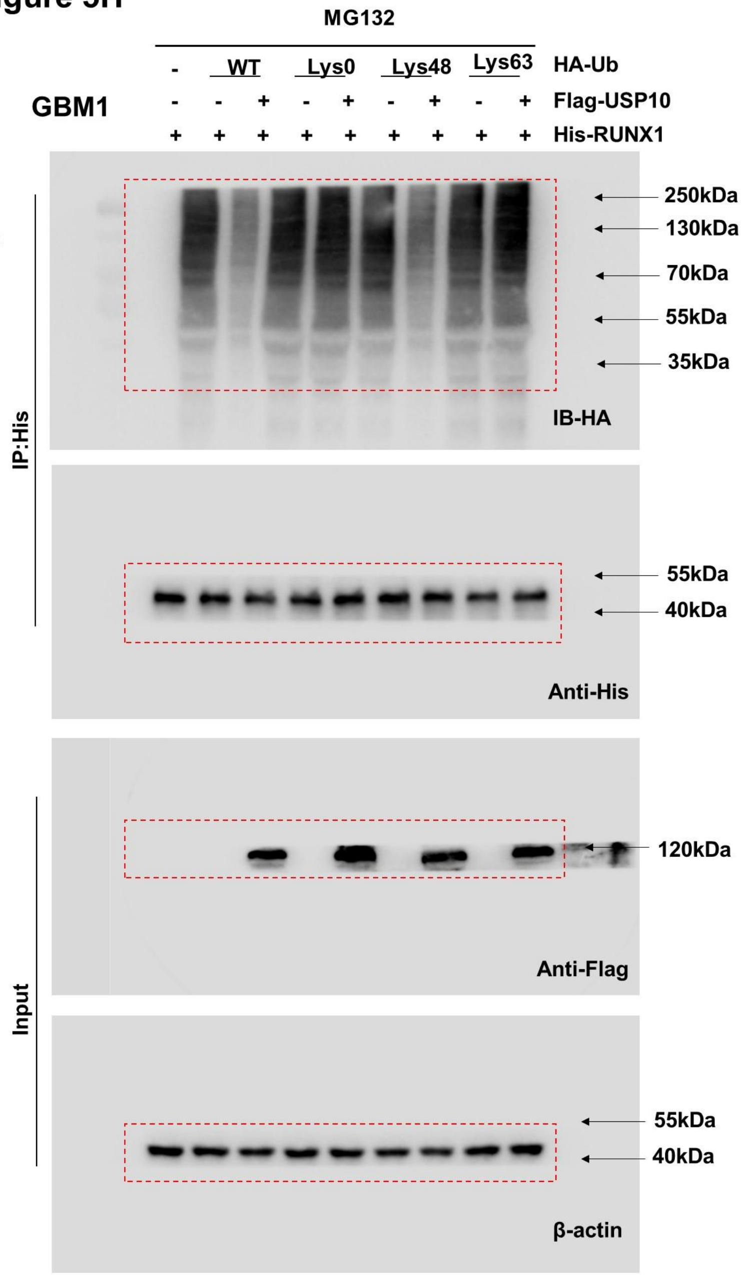

Figure 5I

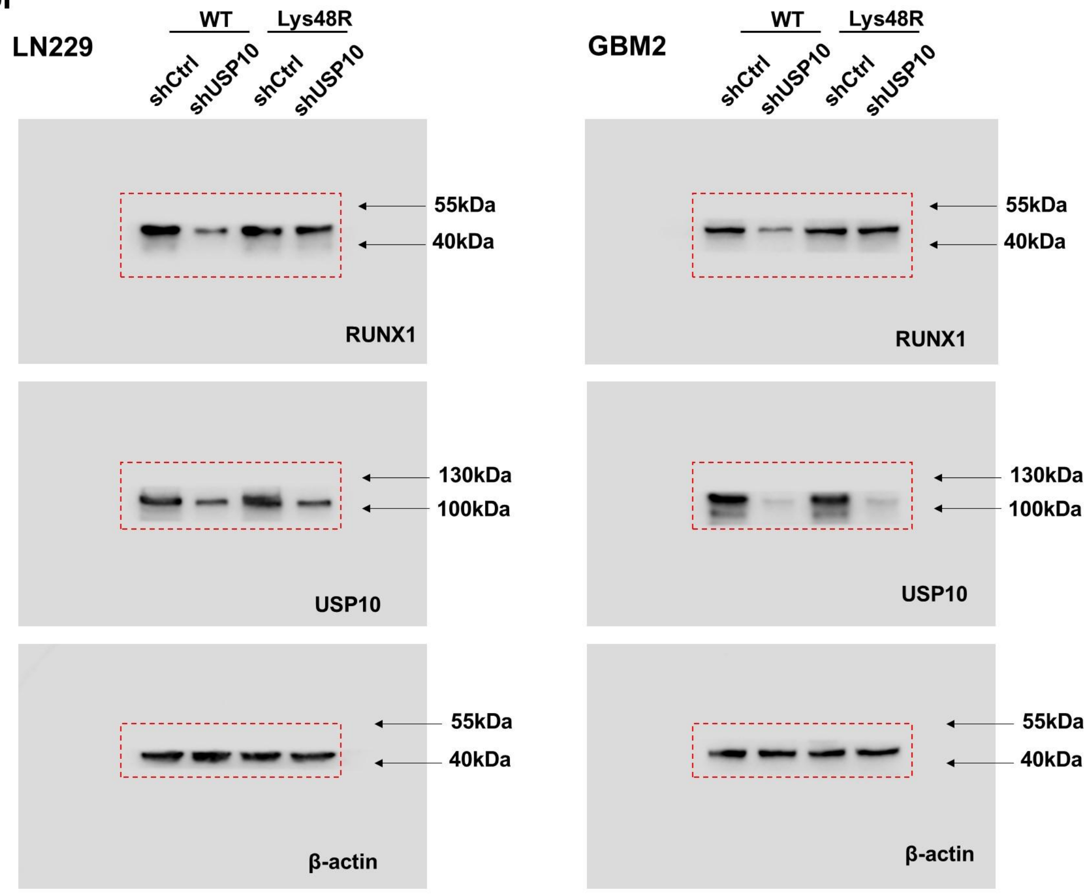
LN229
WT
Lys48R
shCtrl
shUSP10
shCtrl
shUSP10
55kDa
40kDa
RUNX1
130kDa
100kDa
USP10
55kDa
40kDa
β-actin
GBM2
WT
Lys48R
shCtrl
shUSP10
shCtrl
shUSP10
55kDa
40kDa
RUNX1
130kDa
100kDa
USP10
55kDa
40kDa
β-actin

Figure 6A

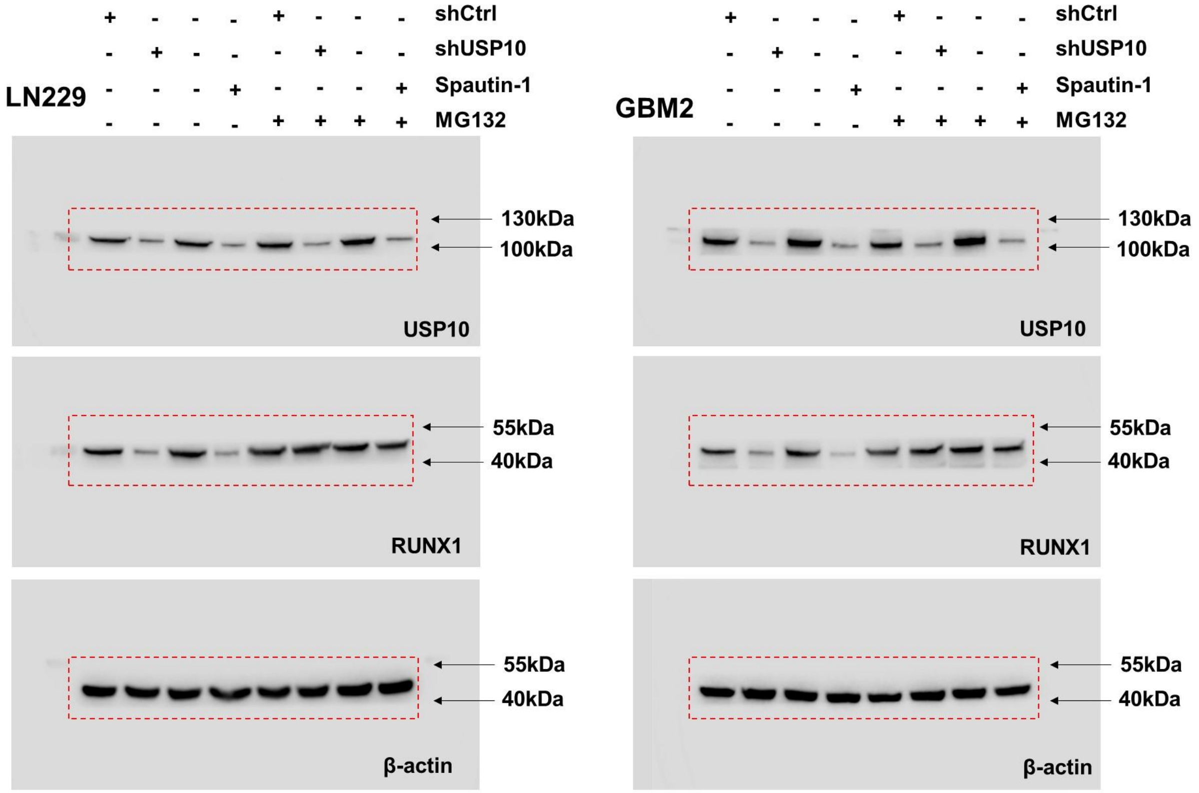

LN229
shCtrl + - - - + - - -
shUSP10 - + - - - + - -
Spautin-1 - - - + - - - +
MG132 - - - - + + + +
130kDa
100kDa
USP10
55kDa
40kDa
RUNX1
55kDa
40kDa
β-actin
GBM2
shCtrl + - - - + - - -
shUSP10 - + - - - + - -
Spautin-1 - - - + - - - +
MG132 - - - - + + + +
130kDa
100kDa
USP10
55kDa
40kDa
RUNX1
55kDa
40kDa
β-actin

Figure 6B

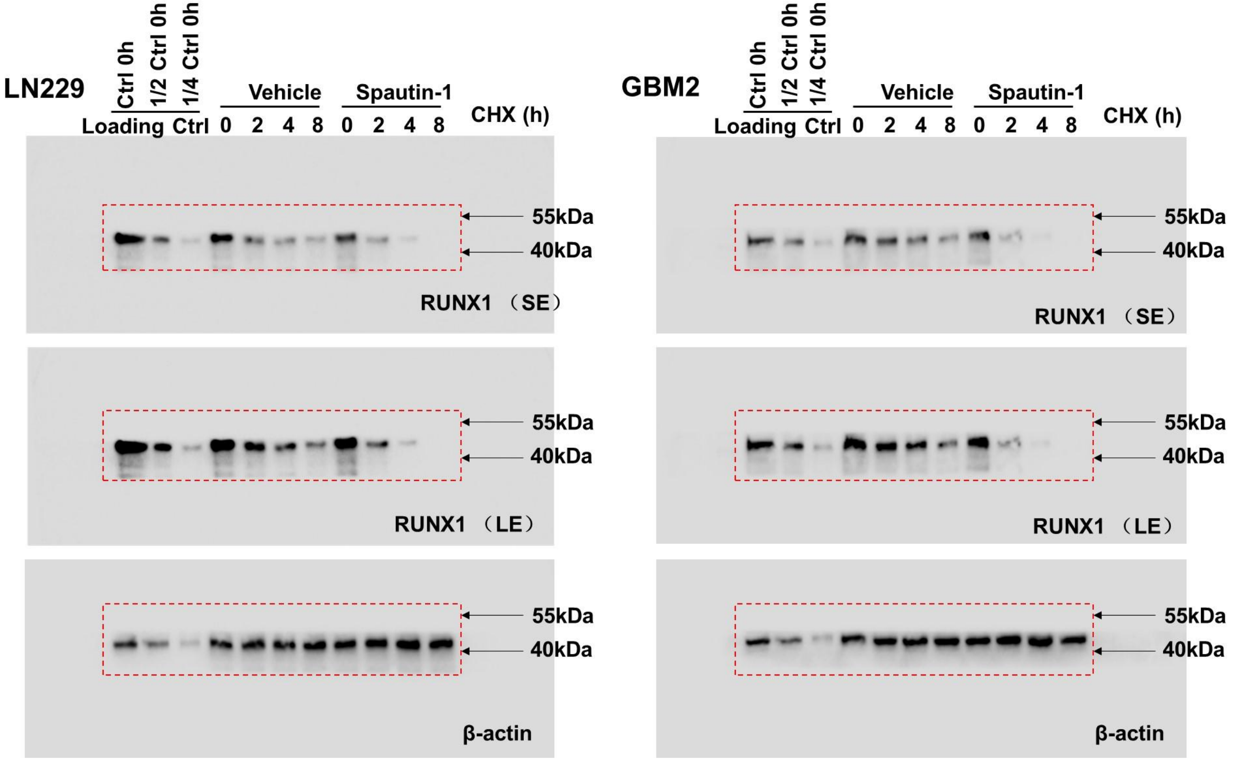
LN229
Ctrl 0h
1/2 Ctrl 0h
1/4 Ctrl 0h
Loading Ctrl
Vehicle
Spautin-1
0 2 4 8 0 2 4 8
CHX (h)
55kDa
40kDa
RUNX1 （SE）
RUNX1 （LE）
β-actin
GBM2
Ctrl 0h
1/2 Ctrl 0h
1/4 Ctrl 0h
Loading Ctrl
Vehicle
Spautin-1
0 2 4 8 0 2 4 8
CHX (h)
55kDa
40kDa
RUNX1 （SE）
RUNX1 （LE）
β-actin

Figure 6C

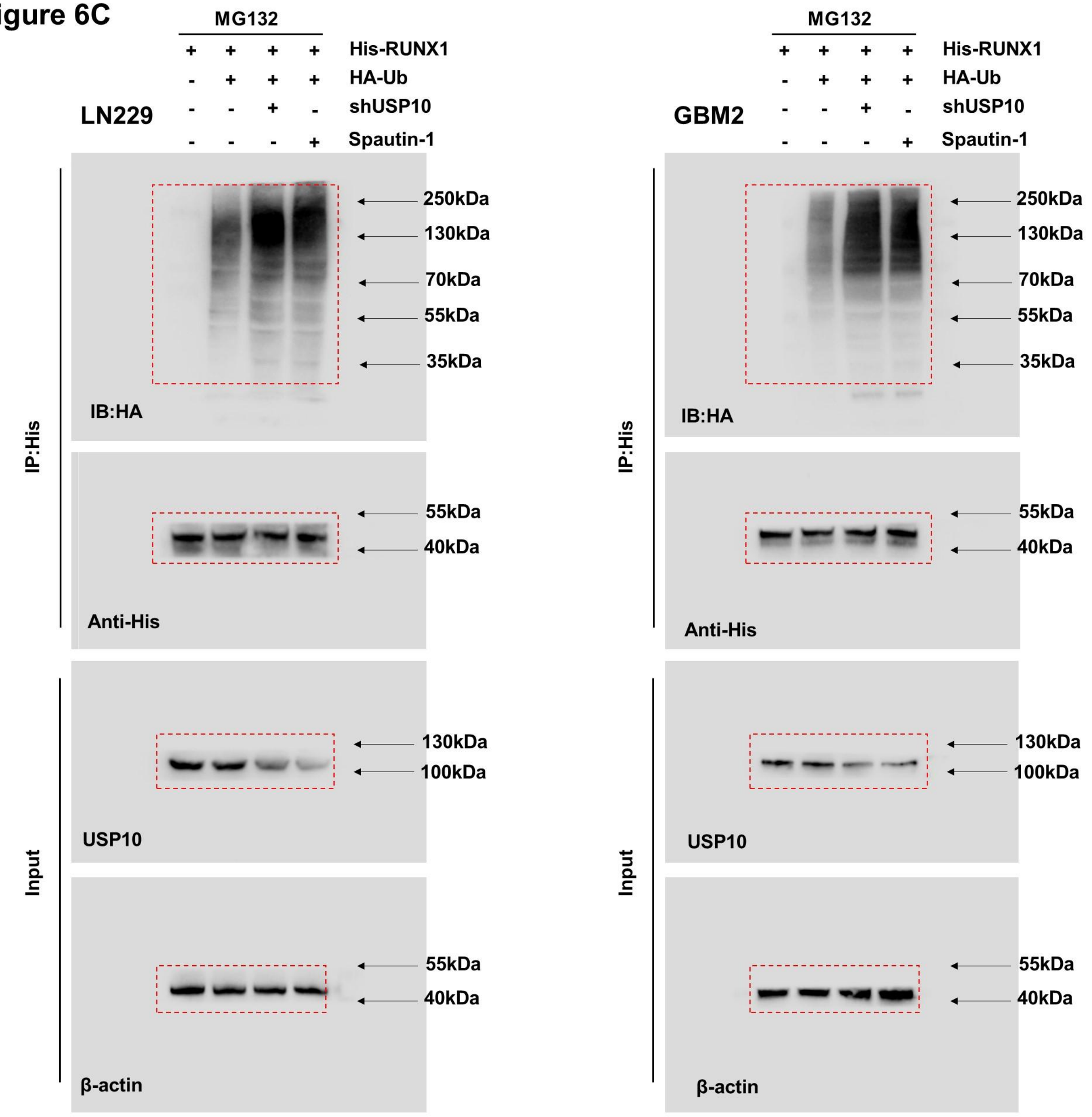
MG132
His-RUNX1
HA-Ub
shUSP10
Spautin-1
LN229
GBM2
IP:His
IB:HA
Anti-His
Input
USP10
β-actin
250kDa
130kDa
100kDa
70kDa
55kDa
40kDa
35kDa

Figure 6D

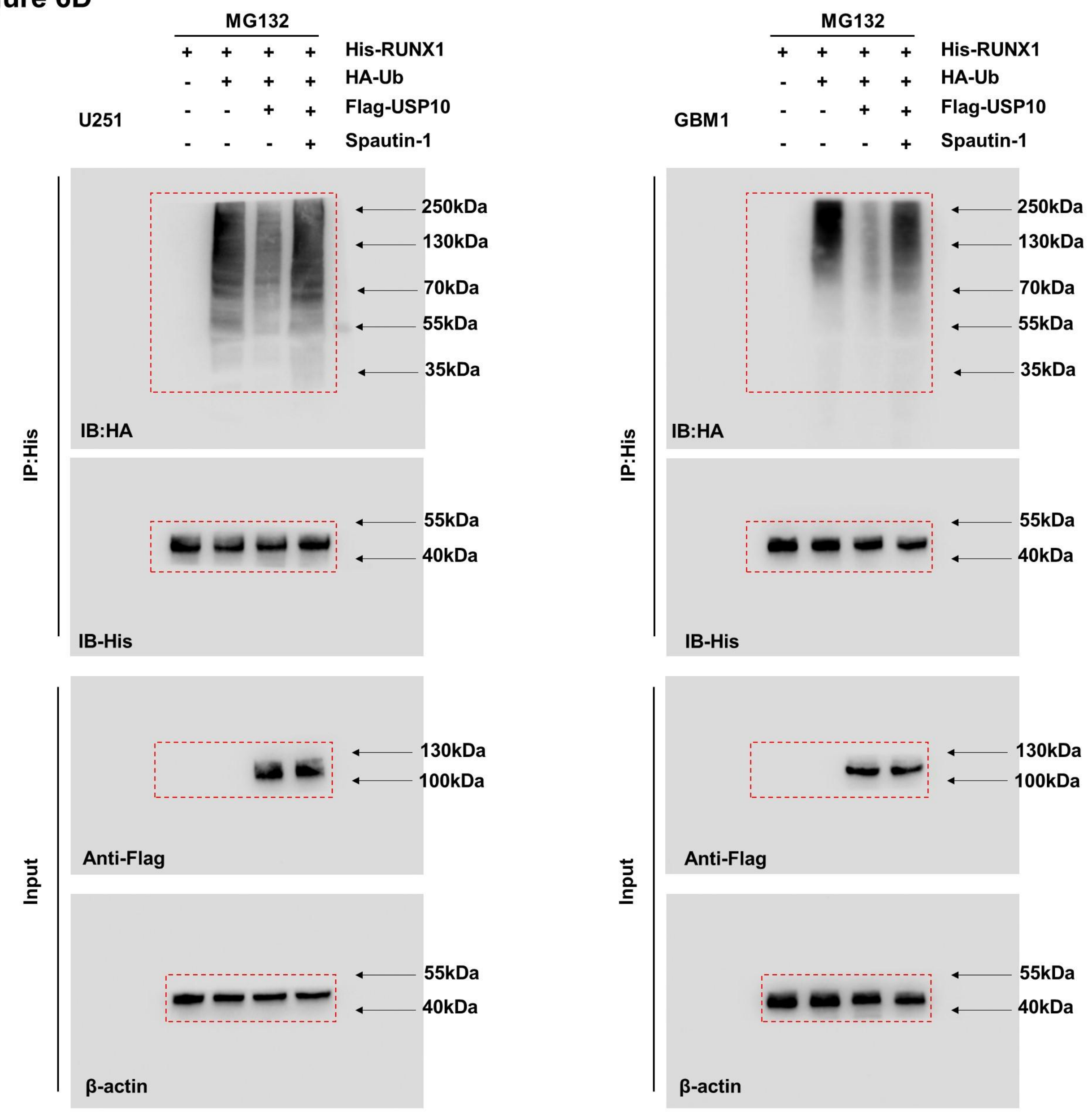
MG132
His-RUNX1
HA-Ub
Flag-USP10
Spautin-1
U251
+ + + +
- + + +
- - + +
- - - +
IP:His
IB:HA
250kDa
130kDa
70kDa
55kDa
35kDa
IB-His
55kDa
40kDa
Input
Anti-Flag
130kDa
100kDa
β-actin
55kDa
40kDa
GBM1
MG132
His-RUNX1
HA-Ub
Flag-USP10
Spautin-1
IP:His
IB:HA
IB-His
Input
Anti-Flag
β-actin

Figure 6E

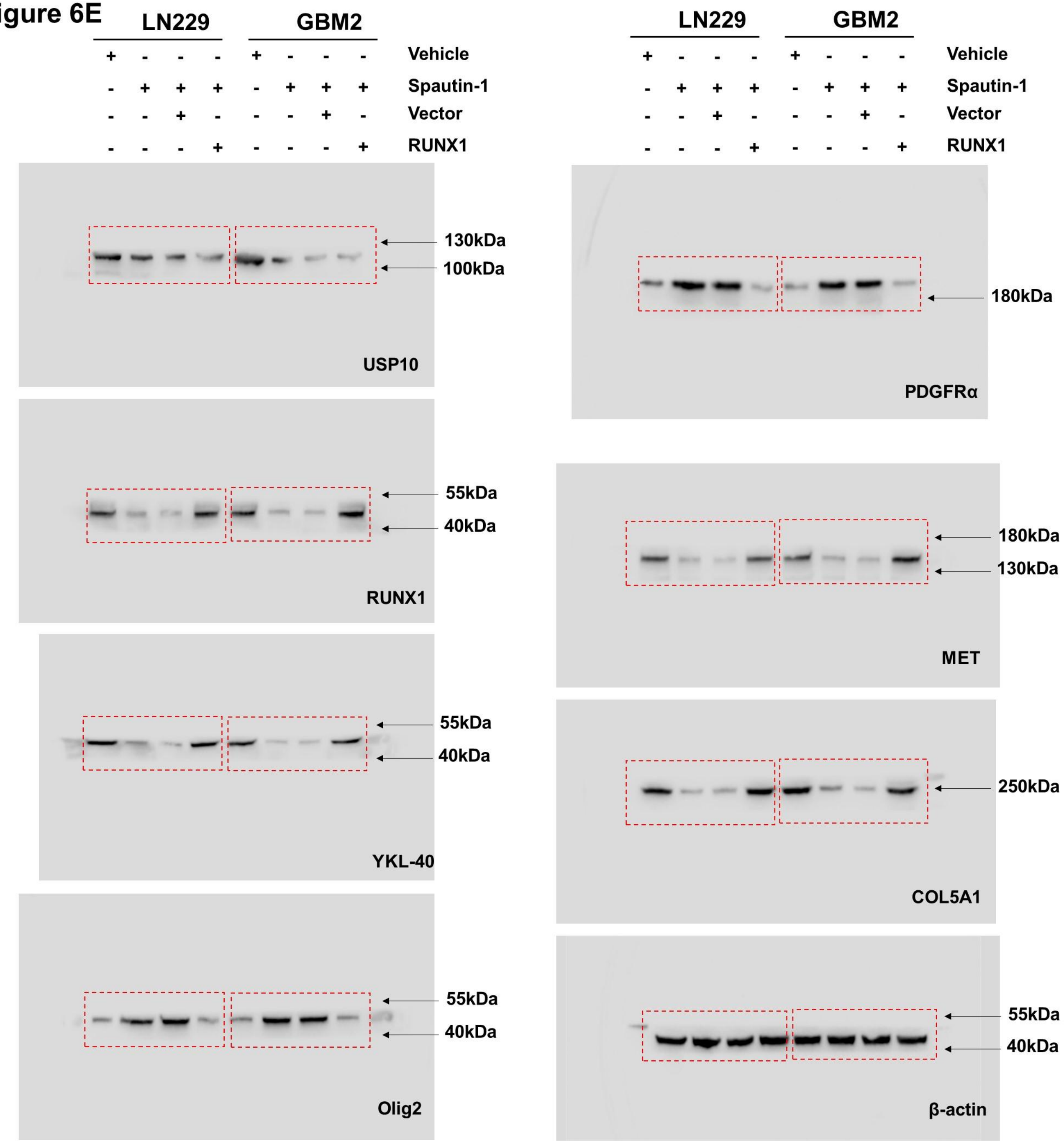
LN229
GBM2
Vehicle
Spautin-1
Vector
RUNX1
130kDa
100kDa
USP10
55kDa
40kDa
RUNX1
55kDa
40kDa
YKL-40
55kDa
40kDa
Olig2
LN229
GBM2
Vehicle
Spautin-1
Vector
RUNX1
180kDa
PDGFRα
180kDa
130kDa
MET
250kDa
COL5A1
55kDa
40kDa
β-actin

Figure 6F

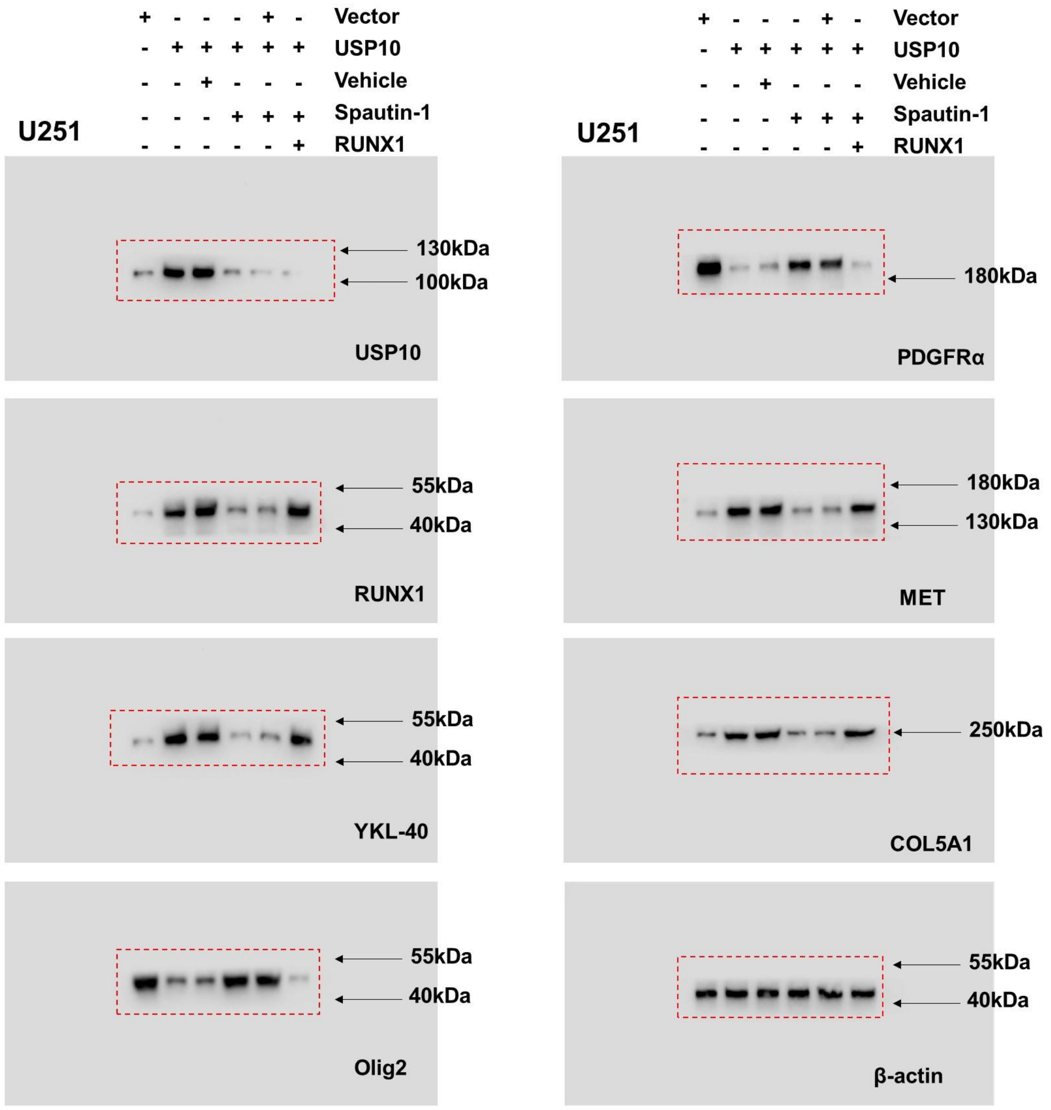
U251
+ - - - + - Vector
- + + + + + USP10
- - + - - - Vehicle
- - - + + + Spautin-1
- - - - - + RUNX1
130kDa
100kDa
USP10
55kDa
40kDa
RUNX1
55kDa
40kDa
YKL-40
55kDa
40kDa
Olig2
U251
+ - - - + - Vector
- + + + + + USP10
- - + - - - Vehicle
- - - + + + Spautin-1
- - - - - + RUNX1
180kDa
PDGFRα
180kDa
130kDa
MET
250kDa
COL5A1
55kDa
40kDa
β-actin

Figure 6F

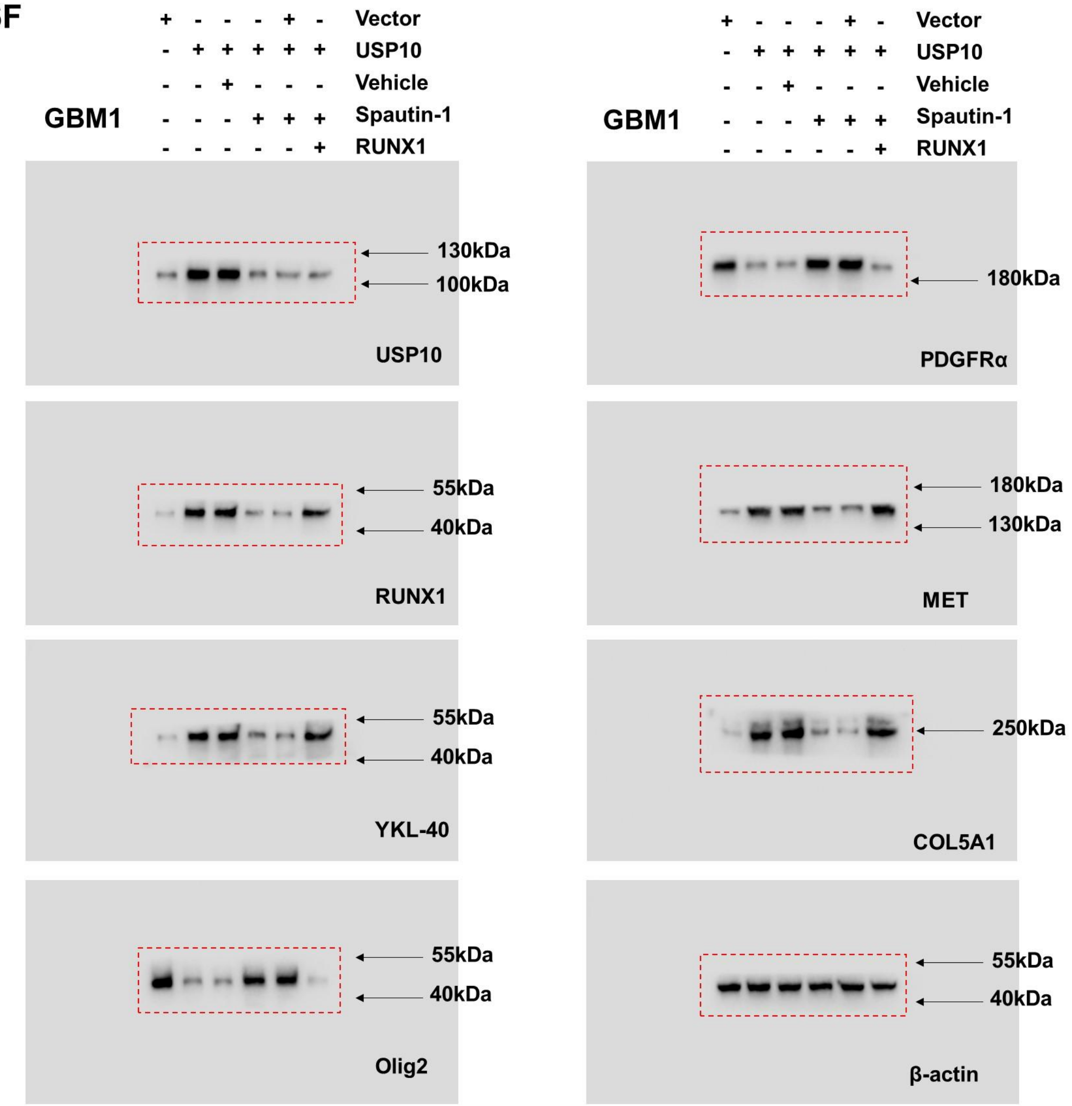
GBM1
+ - - - + - Vector
- + + + + + USP10
- - + - - - Vehicle
- - - + + + Spautin-1
- - - - - + RUNX1
130kDa
100kDa
USP10
55kDa
40kDa
RUNX1
55kDa
40kDa
YKL-40
55kDa
40kDa
Olig2
GBM1
+ - - - + - Vector
- + + + + + USP10
- - + - - - Vehicle
- - - + + + Spautin-1
- - - - - + RUNX1
180kDa
PDGFRα
180kDa
130kDa
MET
250kDa
COL5A1
55kDa
40kDa
β-actin

Figure 7B

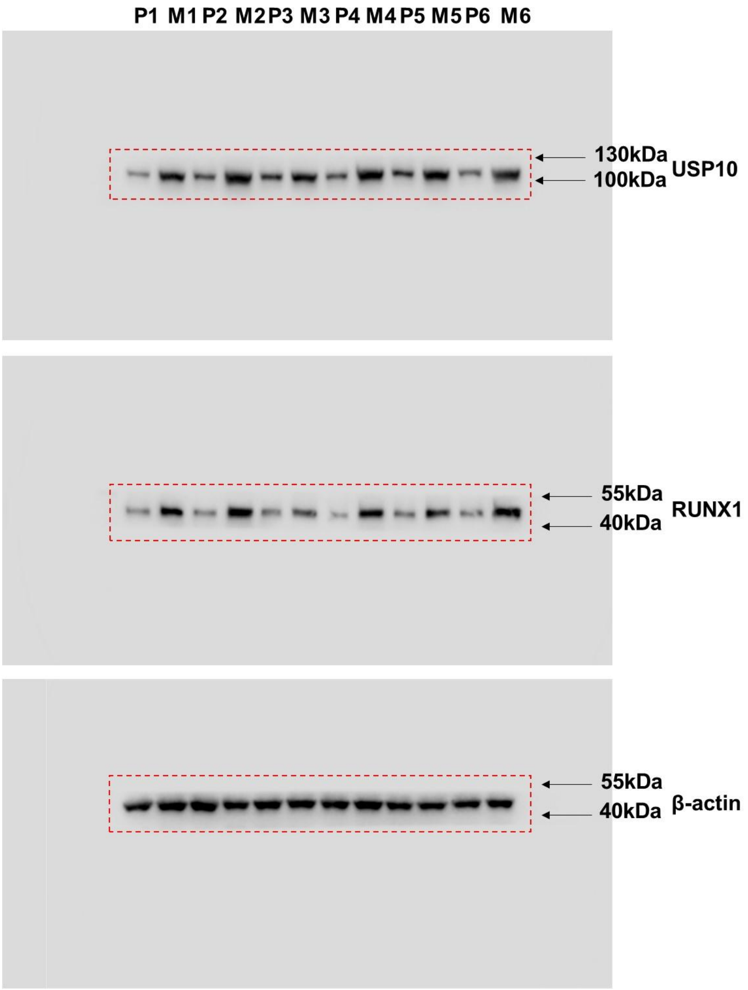

P1 M1 P2 M2 P3 M3 P4 M4 P5 M5 P6 M6
130kDa
100kDa
USP10
55kDa
40kDa
RUNX1
55kDa
40kDa
β-actin

Supplementary Figure 1A

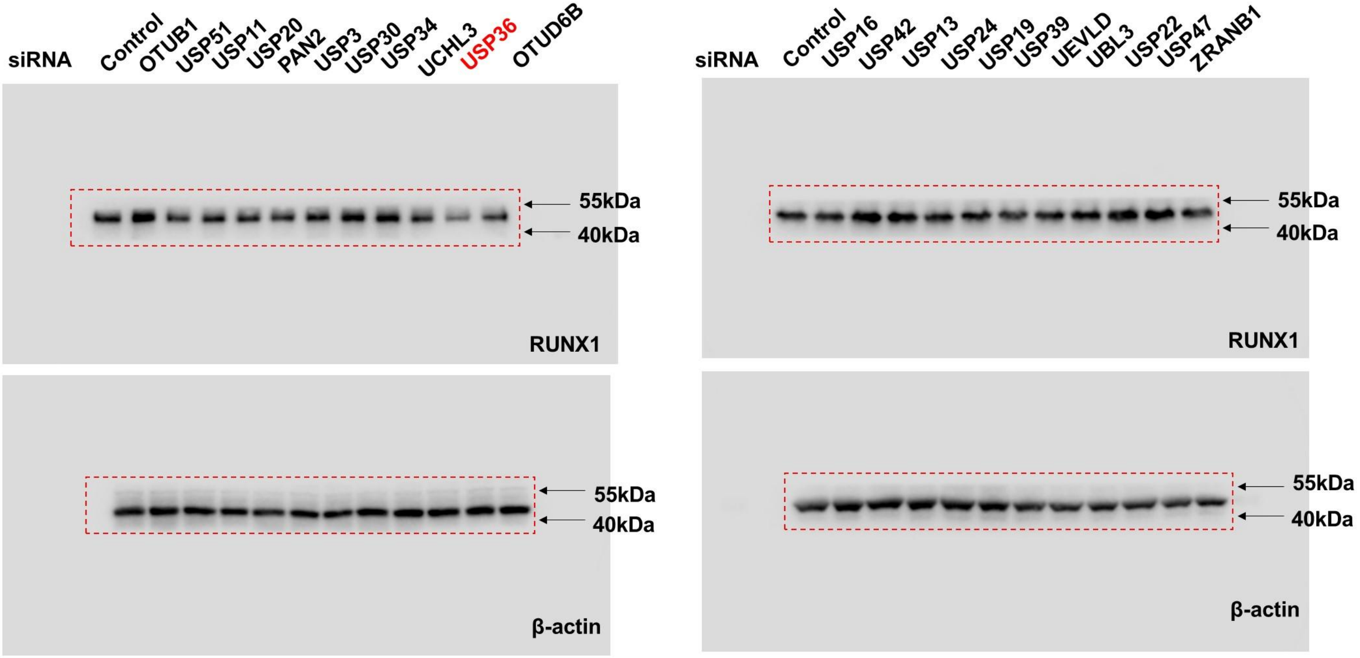

Supplementary Figure 1A

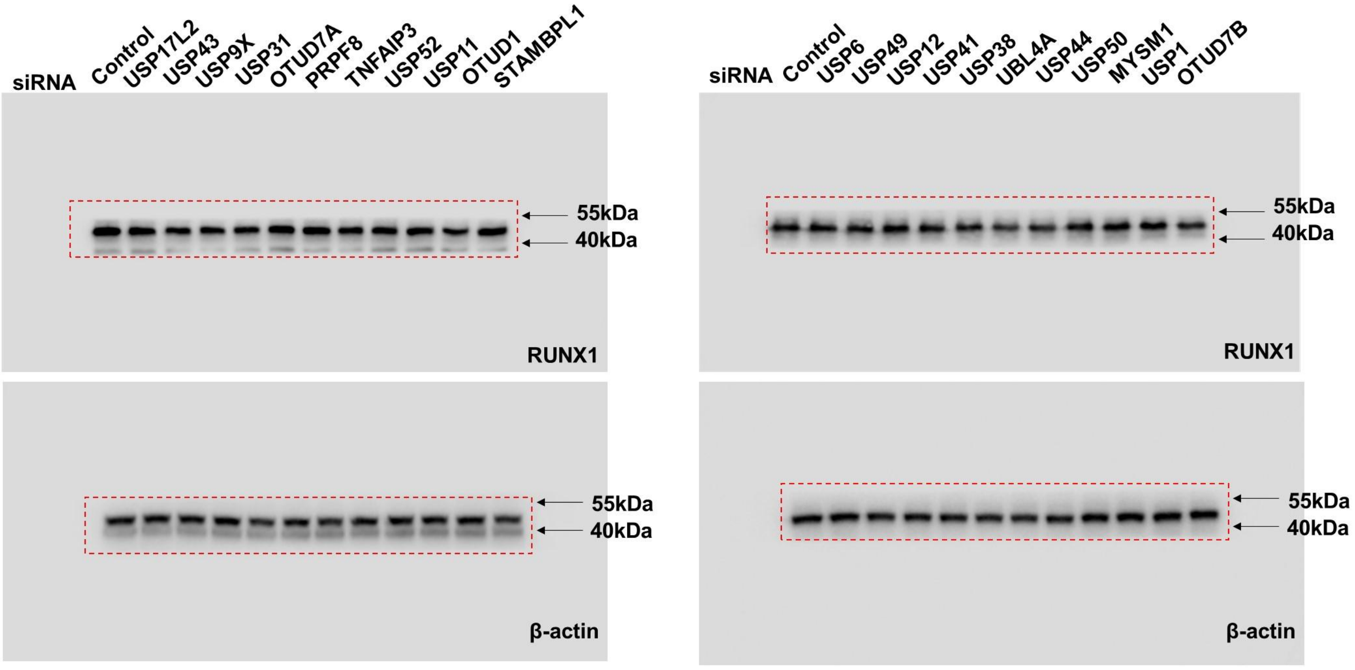

Supplementary Figure 1A

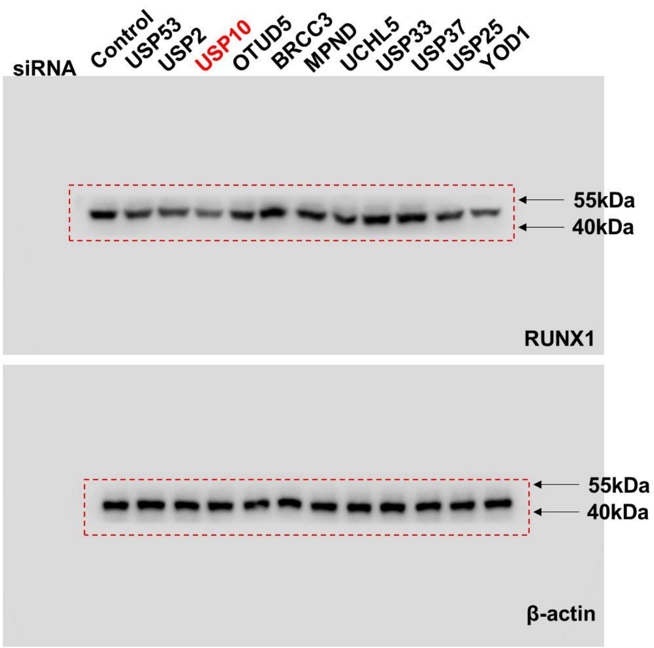

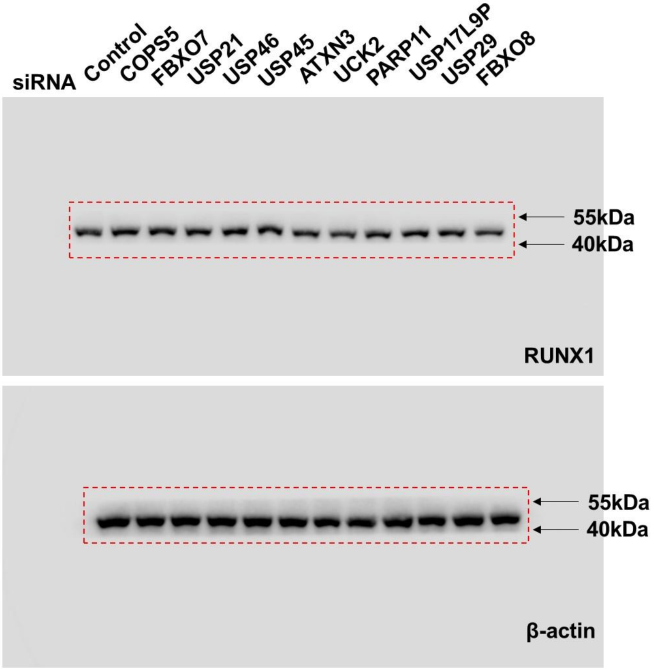

Supplementary Figure 1A

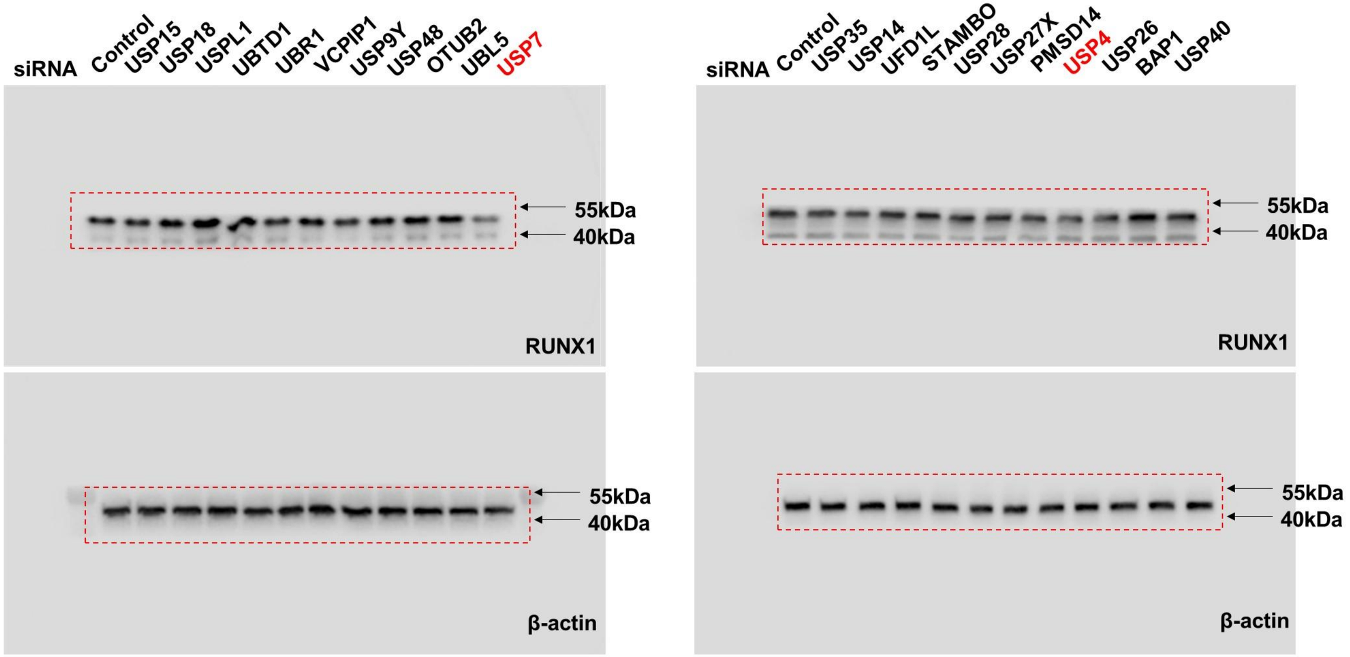

Supplementary Figure 1A

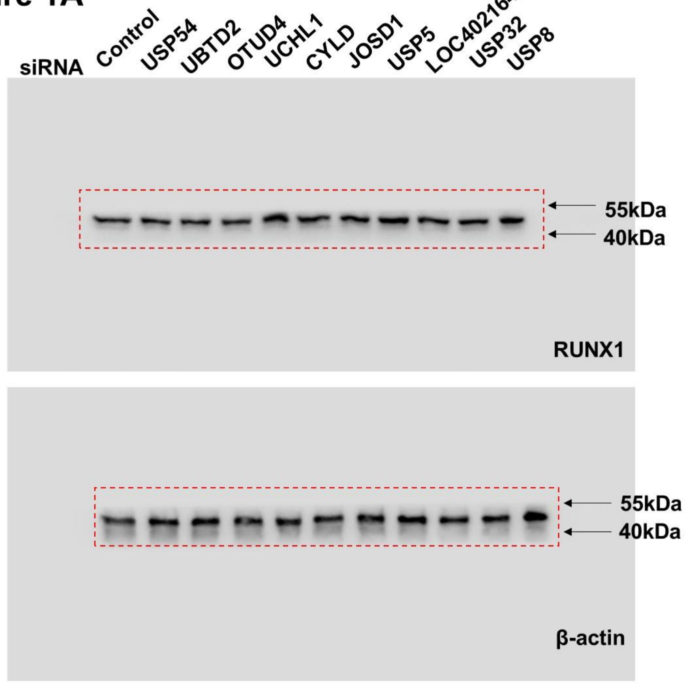

Supplementary Figure 2B

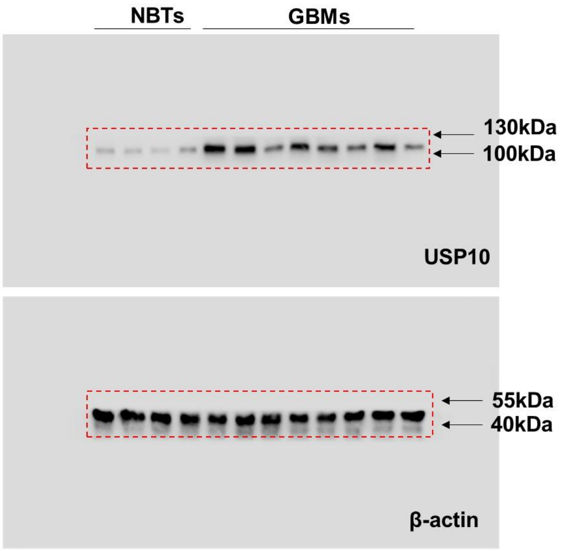

Supplementary Figure 2C

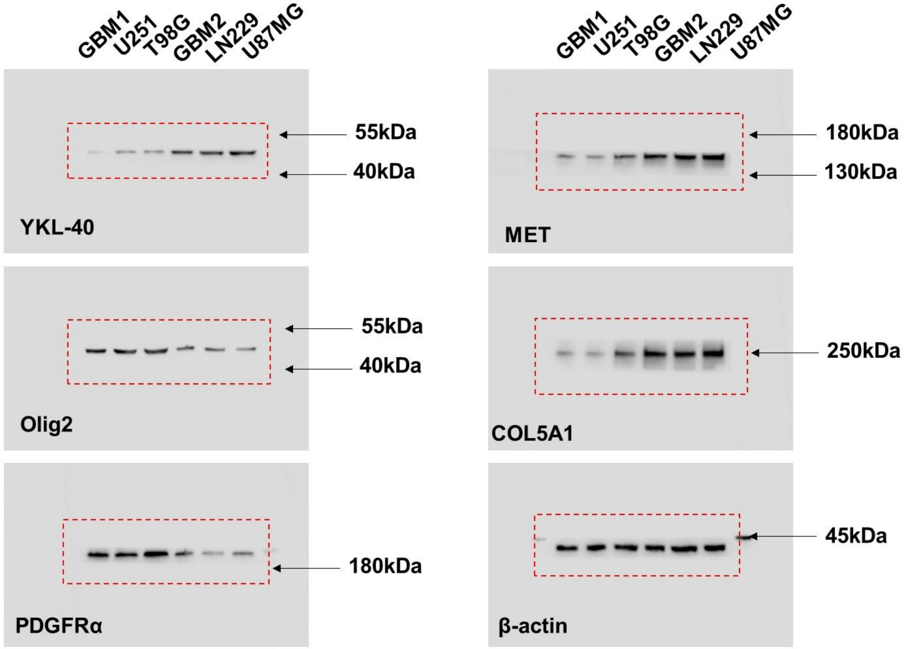

Supplement: Supplementary file 10 — Original images of western blot [file 41419_2023_5734_MOESM10_ESM.pdf]
